# Enhancing fatigue life by ductile-transformable multicomponent B2 precipitates in a high-entropy alloy

**DOI:** 10.1038/s41467-021-23689-6

**Published:** 2021-06-11

**Authors:** Rui Feng, You Rao, Chuhao Liu, Xie Xie, Dunji Yu, Yan Chen, Maryam Ghazisaeidi, Tamas Ungar, Huamiao Wang, Ke An, Peter. K. Liaw

**Affiliations:** 1 Department of Materials Science and Engineering, The University of Tennessee, Knoxville, TN USA; 2 Neutron Scattering Division, Oak Ridge National Laboratory, Oak Ridge, TN USA; 3 Department of Materials Science and Engineering, The Ohio State University, Columbus, OH USA; 4 State Key Laboratory of Mechanical System and Vibration, Shanghai Jiao Tong University, Shanghai, China; 5 Department of Materials Physics, Eötvös University Budapest, Budapest, Hungary

**Keywords:** Mechanical properties, Metals and alloys

## Abstract

Catastrophic accidents caused by fatigue failures often occur in engineering structures. Thus, a fundamental understanding of cyclic-deformation and fatigue-failure mechanisms is critical for the development of fatigue-resistant structural materials. Here we report a high-entropy alloy with enhanced fatigue life by ductile-transformable multicomponent B2 precipitates. Its cyclic-deformation mechanisms are revealed by real-time in-situ neutron diffraction, transmission-electron microscopy, crystal-plasticity modeling, and Monte-Carlo simulations. Multiple cyclic-deformation mechanisms, including dislocation slips, precipitation strengthening, deformation twinning, and reversible martensitic phase transformation, are observed in the studied high-entropy alloy. Its improved fatigue performance at low strain amplitudes, i.e., the high fatigue-crack-initiation resistance, is attributed to the high elasticity, plastic deformability, and martensitic transformation of the B2-strengthening phase. This study shows that fatigue-resistant alloys can be developed by incorporating strengthening ductile-transformable multicomponent intermetallic phases.

## Abstract (web summary)

A fundamental understanding of fatigue-failure mechanisms is key to develop robust structural materials. Here the authors report a high entropy alloy with enhanced fatigue life by ductile transformable multicomponent B2 precipitates, as revealed by combined experimental and simulation methods.

## Introduction

Nearly 90% of mechanical service failures are caused by fatigue at cyclic stresses far below the ultimate or yield strengths of the materials involved^1^. Therefore, fatigue endurance of a structural material is a pivotal criterion to evaluate whether it can be reliably used in practical engineering environments. To improve the fatigue endurance of a material, one of the common methods is to improve its fatigue strength by introducing intermetallic precipitates for hardening^1^. However, a reduced fatigue-crack-initiation resistance as a harmful side effect due to the introduction of additional phase interfaces is always accompanied in this conventional alloy-design wisdom. This trend is particularly obvious when the materials repeatedly undergo low plastic deformation, i.e., low plastic strain amplitudes, such as the case of structural components being constantly impacted. Therefore, the conventional alloy-design strategies encounter the dilemma of improving both fatigue strength and fatigue-crack-initiation resistance. Recently, a new alloy-design concept, called high-entropy alloys (HEAs), shows great potential in enhancing materials’ mechanical performance^2–9^. The diverse characteristics in HEAs, such as severe lattice distortion, multicomponent precipitates, short-range ordering (SRO), and tunable stacking-fault energies (SFE)^10,11^, can be utilized to improve materials fatigue performance^12–18^. Particularly, atypical ductile multicomponent intermetallic phases, recently observed in HEAs and distinct from brittle intermetallics, can enhance the strength without sacrificing too much ductility^7,19,20^. Such an interesting character is believed to affect significantly mechanical behaviors, including the cyclic plastic-deformation behavior that has not been reported yet.

Inspired by this idea, here we design a multicomponent B2 precipitates-strengthened HEA to improve the fatigue performance of structural materials. We find that the designed alloy shows at least four times longer fatigue life than other conventional alloys at a low plastic strain amplitude of ~0.03% by incorporating the ductile-transformable multicomponent B2 precipitates, exhibiting enhanced fatigue-crack-initiation resistance. The underlining mechanisms are revealed by using the state of the art real-time in situ neutron diffraction and advanced electron microscopies together with the crystal-plasticity modeling and Monte-Carlo (MC) simulation. As a result, we show that the design idea by integrating ductile-transformable multicomponent intermetallic precipitates and providing various beneficial cyclic-deformation mechanisms leads to a new direction of designing advanced fatigue-resistant alloys.

## Results

### Microstructures

**Fig. 1:**
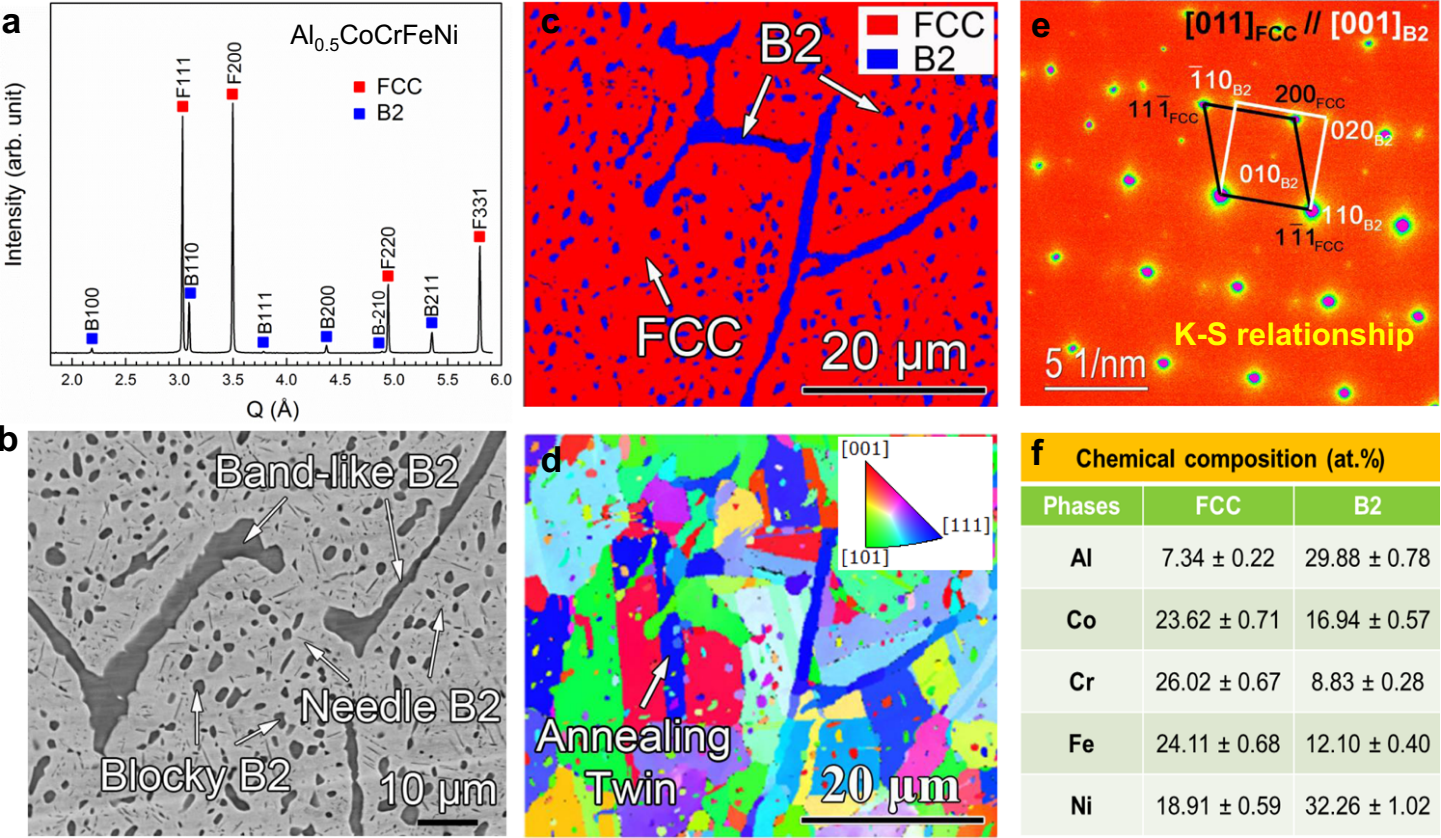
Phase and microstructural information of the studied HEA. **a** HEXRD of the Al_0.5_CoCrFeNi alloy, showing the presence of FCC and B2 phases (the diffraction peaks of FCC and B2 phases are indexed as F-hkl and B-hkl, respectively). **b** Back-scattering electron (BSE) image of the studied alloy, presenting the three different morphologies of the B2 phase. **c**, **d** EBSD-phase map and corresponding grain-orientation image, respectively. **e** SAED patterns of FCC and B2 phases along FCC’s [011] and B2’s [001] directions, indicating the K–S crystallographic relationship between the FCC matrix and the B2 precipitates. **f** Chemical compositions of the FCC and B2 phases determined by EDS. The errors are determined based on EDS counting statistics.

The studied Al_0.5_CoCrFeNi alloy has the face-centered cubic (FCC) matrix and the multicomponent B2 strengthening precipitates verified by the high-energy X-ray diffraction (HEXRD) (Fig. 1a) and electron-backscattered diffraction (EBSD) phase map (Fig. 1c). The lattice parameters of FCC (3.593 Å) and B2 (2.874 Å) phases were determined from the Rietveld refinement of the HEXRD data. The refined phase fraction of the B2 phase (~12%) is consistent with the EBSD-phase-map measurement. The B2 precipitates exhibit three different hierarchical morphologies, i.e., the fine and dense B2 precipitates with blocky and needle shapes within the FCC grains and the coarse band-like B2 phase located at the parent grain boundaries (Fig. 1b, c). The FCC phase has a nominal grain size of ~9 μm (Fig. 1d). Fig. 1e shows the selected-area electron diffraction (SAED) pattern along FCC’s [011] and B2’s [001] zone axes. It is noted that the FCC matrix and the B2 precipitates satisfy the Kurdjumov–Sachs (K–S) crystallographic relationship, i.e., {111}_FCC_//{110}_B2_; <110>_FCC_// <111> _B2_. The chemical compositions of the FCC and B2 phases determined by energy-dispersive spectroscopy (EDS) are given in Fig. 1f and Supplementary Fig. 1. Correspondingly, the configuration entropies of the FCC and B2 phases were calculated as 1.54 R and 1.5 R, respectively, which meet the HEAs’ definition^2,10^. The B2 phase is rich in Ni and Al elements, indicative of the NiAl-type B2 phase.

### Tensile and fatigue properties

**Fig. 2:**
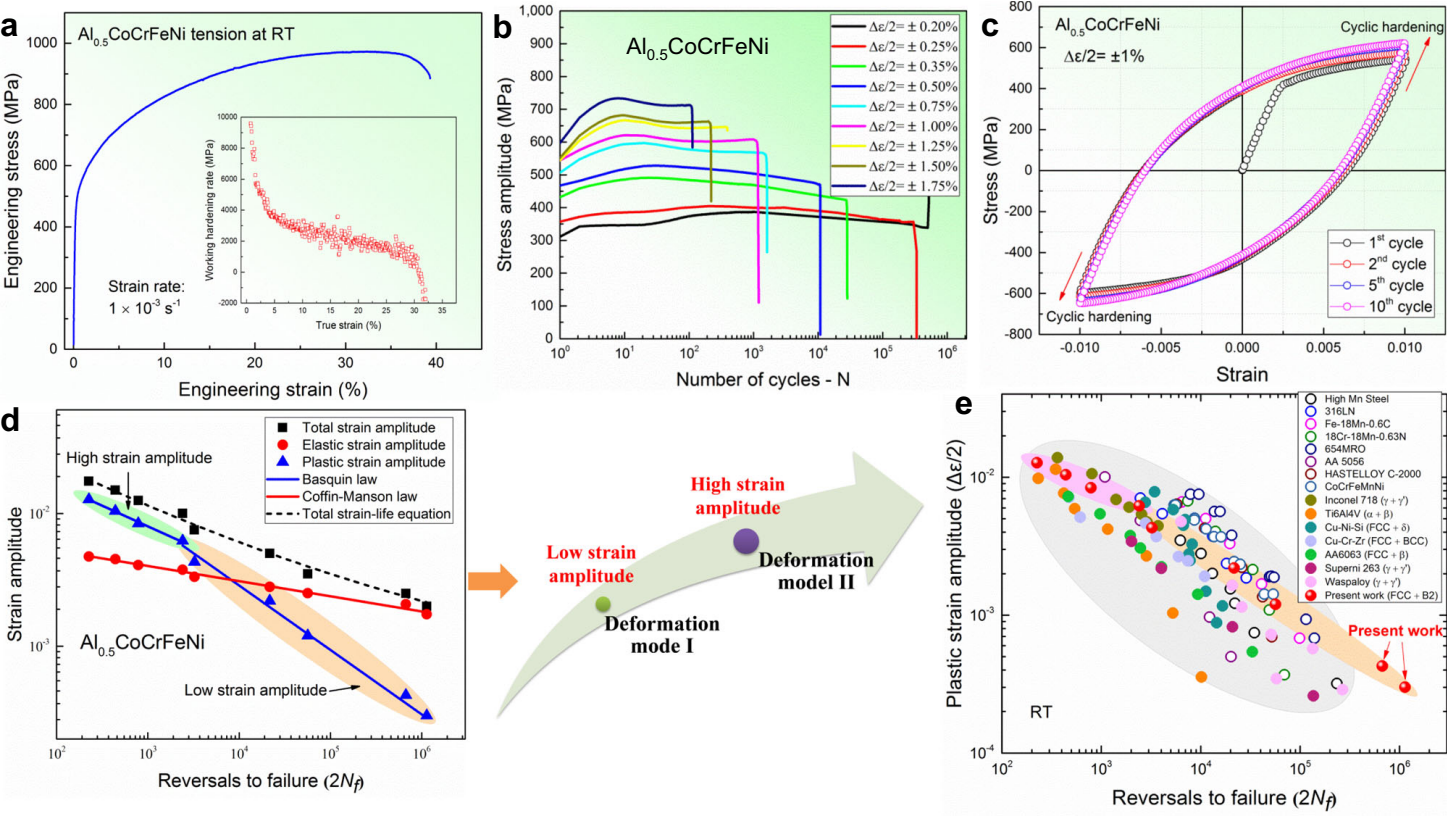
Tensile and LCF results of the Al_0.5_CoCrFeNi alloy. **a** Uniaxial tensile curve of the Al_0.5_CoCrFeNi alloy at RT (an inset figure shows its working-hardening rate versus true strain). **b** Cyclic stress response (CSR) curves of the Al_0.5_CoCrFeNi HEA at different strain amplitudes. **c** Hysteresis loops of the Al_0.5_CoCrFeNi HEA at selected numbers of cycles fatigued at the strain amplitude of ±1%. **d** Total strain amplitude, elastic-strain amplitude, and plastic-strain amplitude versus the number of reversals to failure (2*N*_*f*_), displaying the presence of a bilinear Coffin–Manson relationship that signifies a variation of the cyclic-deformation modes (cartoon figure). **e** The comparison of Coffin–Manson fatigue data for the present alloy and other conventional alloys, showing the superior LCF properties of the studied HEA at low strain amplitudes, relative to other conventional alloys (open-circle and solid-circle symbols represent single-phase and precipitation-strengthened alloys, respectively)^24–37^.

A monotonic uniaxial tension test shows that this alloy has a good combination of the yield strength (493 ± 4 MPa), work hardening (>1000 MPa throughout the uniform deformation), and ultimate tensile stress of 973 ± 25 MPa with the ductility up to 40 ± 2% (Fig. 2a). Regarding the cyclic performance, Fig. 2b presents the evolution of stress amplitudes with the number of cycles under different strain amplitudes. Obviously, the cyclic stress response (CSR) and fatigue life depend on the applied strain amplitudes. As the strain amplitude increases, the stress amplitude increases, but the fatigue life decreases. Moreover, changing from cyclic hardening to softening, and/or saturation was also observed with increasing fatigue cycles. The stress amplitude increased rapidly in the initial stage of cycling, followed by cyclic softening and/or saturation. The selected hysteresis loops of the strain amplitude of ±1% depict the obvious initial cyclic hardening (Fig. 2c and Supplementary Fig. 2). At larger strain amplitudes (≥ ±1%), a slight cyclic softening was observed after the initial hardening, and then, a cyclic saturation was reached until fracture. However, an obvious cyclic softening happened until fracture when the applied strain amplitude was smaller than ±1% (Fig. 2b).

The fatigue life of this HEA was evaluated by the strain/stress-life equations, i.e., Basquin and Coffin–Manson laws (Supplementary Note 1 and Supplementary Table 1)^21–23^. Correspondingly, the relationship of the total strain amplitude, elastic-strain amplitude, and plastic strain amplitude as a function of the number of reversals to failure (2*N*_*f*_) is plotted in Fig. 2d. It is worth noting that a bilinear Coffin–Manson rule was observed in the relationship of the fatigue life versus plastic strain amplitude, suggesting a transition of the cyclic-deformation mode with the applied strain amplitudes, as depicted by the cartoon in Fig. 2d. Fig. 2e shows the comparison of the relationship of a reversal to failure (2*N*_*f*_) versus plastic strain amplitude that excluded the contribution of the elastic strain due to different material stiffness for the present alloy and other conventional alloys^24–37^. At high plastic strain amplitudes (>10^−3^), the studied HEA shows comparable low-cycle fatigue (LCF) properties with other traditional materials. However, at low plastic strain amplitudes (<10^−3^), the studied HEA exhibits a much longer fatigue life than those materials, as reflected by the smaller slope of plastic strain amplitude versus 2*N*_*f*_. Most pointedly, compared to other traditional precipitation-strengthened alloys, the multicomponent B2 precipitates-strengthened HEA exhibits the outperforming LCF resistance at low strain amplitudes. For example, at the plastic strain amplitude of ~0.03% level, the current HEA shows at least four times longer life than conventional alloys.

### In situ neutron-diffraction studies

**Fig. 3:**
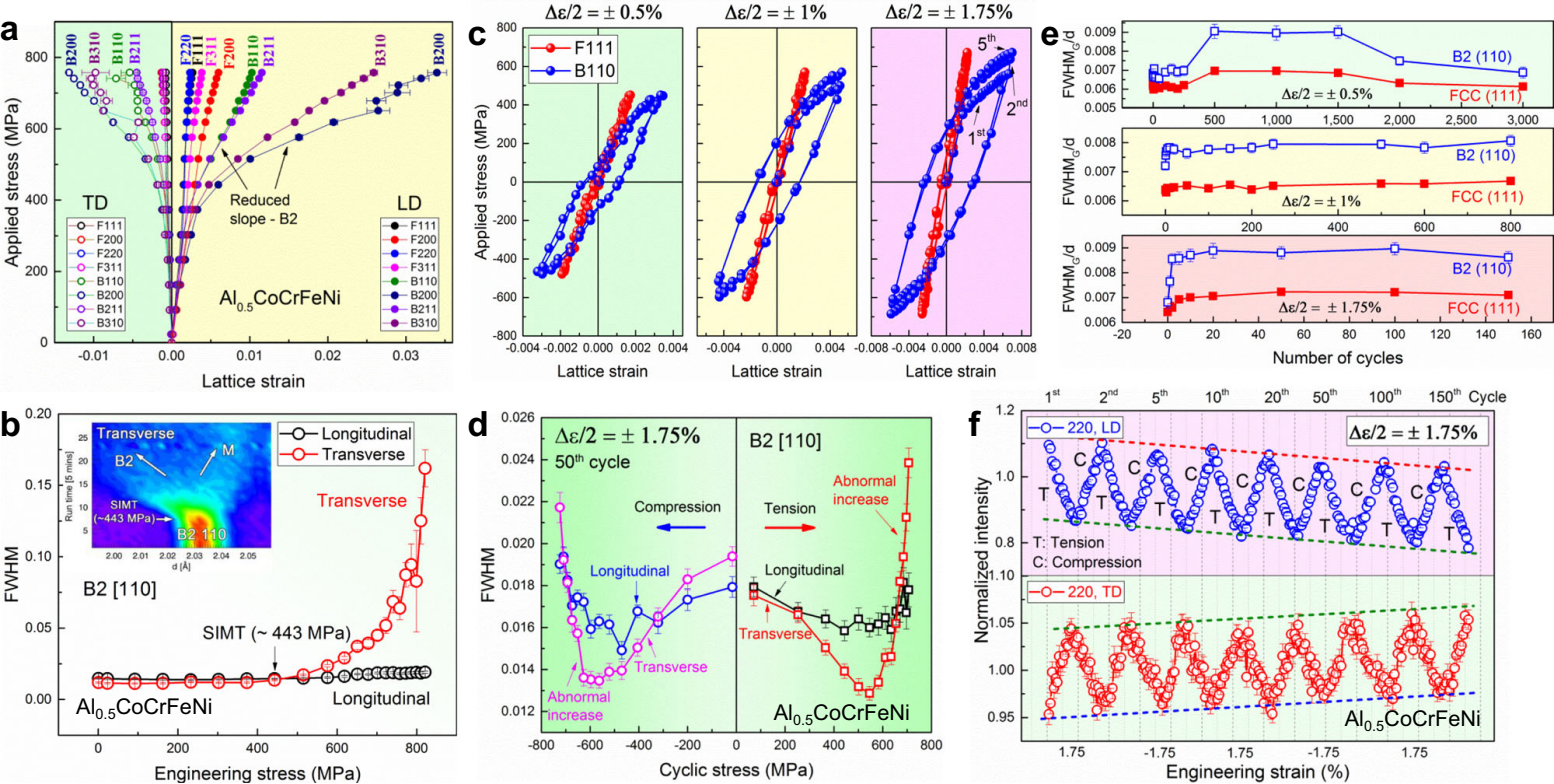
Real-time in situ neutron-diffraction results. **a** Lattice strain as a function of applied stress during the uniaxial tension. **b** The evolution of FWHM as a function of stress along the transverse direction during tension [The inset is a two-dimensional (2D) contour map showing the evolution of the B2-{110} d-spacing], indicating the presence of martensitic transformation. **c** Lattice-strain evolutions of the FCC-{111} and B2-{110} at selected first, second, and fifth cycles along the loading direction. **d** The relationship of the B2-[110] FWHM versus applied stress along longitudinal and transverse directions at the 50th cycle with the strain amplitude of ±1.75%. **e** The evolution of FWHM_G_/d versus the number of cycles at the strain amplitudes of ±0.5%, ±1%, and ±1.75%. **f** In situ neutron diffraction peak-intensity evolution at different fatigue cycles along longitudinal and transverse directions at the strain amplitude of ±1.75%. The error bars in all the figures are obtained from the uncertainties of the single-peak fitting on *hkl* diffraction peaks.

The fatigue performance of the precipitates-hardened alloy was unraveled by real-time in situ neutron diffractions. Fig. 3a presents the lattice strain versus applied tensile stress along the longitudinal and transverse directions (LD and TD) for the selected orientations in both FCC and B2 phases, respectively. A linear response of the lattice strain to the applied stress is found during the elastic range. Different slopes among various grain families and phases are due to the elastic anisotropy. In the FCC phase, the grain subset with a diffraction peak of {200} along the LD is the hardest among the grain subsets investigated, while {111} and {220} grain subsets are soft. Interestingly, the lattice strains in the B2 phase are significantly larger (nearly ten times) than those of the FCC phase, especially the B2-{200} and {310} grains. It manifests that the B2 phase continuously bears more stress after yielding. A similar load partition was also observed during the cyclic loading (Fig. 3c). As the cyclic strain amplitude increases, more energy is dissipated in both the B2 and FCC phases, as evidenced by the increased area of the hysteresis loop (Fig. 3c).

Fig. 3b exhibits the evolution of the peak full width at half maximum (FWHM after subtracting the instrument contribution) of B2-{110} as a function of stress along LD and TD during tension. The FWHM of B2-{110} along TD increased significantly when the stress exceeded about 443 MPa (near the yield strength), but the FWHM along LD did not increase so much. The abnormal increase of FWHM along TD is due to the stress-induced martensitic transformation (SIMT), rather than a simple dislocation evolution, as further evidenced by the separation of the B2-{110} peak along TD (the inset figure in Fig. 3b). This phase-transformation behavior was also evidenced by in situ HEXRD patterns (Supplementary Fig. 3). Obviously, this SIMT shows a preferred orientation, which is B2-{110}//TD, similar to a previous report^38^. The SIMT causes the overlap of the peaks belonging to B2 and new martensite phases, leading to the abnormal peak broadening of the B2-{110} peak along TD. During cyclic loading, similar abnormal peak broadening was also observed in the selected cycles corresponding to the strain amplitudes of 1.75 and 1% (Fig. 3d and Supplementary Fig. 4a), but not under the strain amplitude of 0.5% (Supplementary Fig. 4b). It suggests that SIMT obviously occurs at high strain amplitudes, but may happen at low strain amplitudes where the volume fraction of the newly-formed martensite could be too low to be detected during in situ neutron diffraction. Indeed, the SIMT occurs at low strain amplitudes, as evidenced by the ex situ HEXRD patterns (Supplementary Fig. 5b, c). Since the neutron-diffraction signal was not good enough for revealing the martensite structure, we used the synchrotron diffraction range where the peak separation is obvious to determine the martensite’s structure (Supplementary Fig. 6). The martensite phase kept a crystallographic relationship with the parent B2 phase, i.e., B-(110)//M-(020), B-(002)//M-(200), and B-$(\bar{1}10)$//M-(001)^38^. The lattice parameters of the newly-formed martensite were determined to be *a* = 2.831 Å, *b* = 4.076 Å, and *c* = 4.141 Å, an orthorhombic structure.

The dynamic evolution of the dislocation density is qualitatively established by analyzing the FWHM to understand the cyclic hardening and softening behavior in CSR. FWHM is related to the dislocation density by the proportional relation^39^ of

1 $$\rho \propto \frac{{\mathrm{FWHM}}_{G,{\mathrm{hkl}}}^{2}}{{d}_{{\mathrm{hkl}}}^{2}b{C}_{r}}$$

where *ρ* is the total density of randomly-distributed dislocations, hkl is the Miller index, FWHM_G_ is the Gaussian component of the FWHM of the peak obtained from the single-peak fitting by the pseudo-Voigt function in the general structure analysis system (GSAS)^40^, *d* is the lattice-plane spacing, *b* is the magnitude of the Burgers vector, and *C*_*r*_ is the contrast factor related to the dislocation type (edge or screw)^41^.

The representative diffraction peaks of FCC’s {111} and B2’s {110} along LD (not broadened by SIMT) were used to extract their corresponding FWHM, respectively. All the FWHMs were obtained in the load-free condition during cyclic loading. Fig. 3e shows the evolution of FWHM_G_/*d* of the {111} peak of the FCC phase and the {110} peak of the B2 phase at the strain amplitudes of 0.5, 1, and 1.75%. At the low strain amplitude of 0.5%, FWHM_G_/*d* of the FCC-{111} and B2-{110} peaks exhibited a stable stage at the first 250 cycles, and then, quickly increased to a higher level until 500 cycles. Later, a steady stage appeared from the 500th cycle to the 1500th cycle, and finally with a decrease until the 3000th cycle. It indicates that three obvious cyclic stages were present at the strain amplitude of 0.5%: (i) the dislocation rapid multiplication—cyclic hardening; (ii) the balance between the dislocation multiplication and annihilation—cyclic saturation; and (iii) the reduced dislocation density—cyclic softening. In contrast, at higher strain amplitudes of 1 and 1.75%, a rapid increase of FWHM_G_/*d* happened in the first few cycles, then followed by a steady stage. No obvious decrease of FWHM_G_/*d* was found at the later stages, indicating the absence of obvious cyclic softening at high strain amplitudes (≥1%). Note that similar trends of dislocation-density evolutions are also seen in the convolutional-multiple-whole-profile (CMWP) profile analysis^39^ at the strain amplitude of 1% (Supplementary Note 2 and Supplementary Fig. 7). Since the whole-profile analysis requires high-quality diffraction patterns, we only evaluate the dislocation activity using the relationship of FWHM_G_/*d* versus cycles, considering that not good enough diffraction signal during real-time loading. Interestingly, the B2 phase is plastically deformable at all the three strain amplitudes, as demonstrated by the greatly increased FWHM_G_/*d*, which is caused by the stored dislocations, as presented by later TEM observations. The plastically-deformable B2 phase implies that stress relaxation and strain partitioning occur between the FCC matrix and B2 precipitates, which can relieve the stress concentration during cyclic loading. The real-time monitored cyclic-response behaviors at the strain amplitudes of 0.5, 1, and 1.75% are consistent with their corresponding CSRs in Fig. 2b.

Twinning micromechanical behavior in the FCC phase during LCF was also dynamically detected by in situ neutron diffraction^42,43^. In an FCC structure, <111>, <220>, and <331> orientations favor the deformation twinning. Here the <220> orientation is selected to probe the twinning behavior. Fig. 3f shows the evolutions of the normalized intensity versus cycles along LD and TD at the strain amplitudes of 1.75%. In the <220>//LD orientation, both the dislocation slip and twinning decrease the <220> intensity in tension^42,44^. In Fig. 3f, we observed the gradually-decreased peak intensity in tension (highlighted by the red arrows) with the increase of cycles at the strain amplitude of 1.75%, but not in the case at the strain amplitudes of 1% (Supplementary Fig. 8). This trend is due to the extra contribution from deformation twinning as a result of the higher stress amplitude at the strain amplitude of 1.75% that leads to the peak intensity in tension more negative as the cycle increases. Thus, it indicates that the deformation twinning occurred at the high strain amplitude of 1.75%, but not at the intermediate strain amplitudes, 1%. The real-time captured twinning behavior is directly supported by later TEM characterizations.

### Crystal-plasticity modeling

An elastic viscoplastic self-consistent (EVPSC) model incorporated with the martensitic transformation predicted well the internal strain evolution, macroscopic stress–strain curves, and phase-transformation behavior under both monotonic and cyclic deformations (Supplementary Fig. 9 and Supplementary Table 2). The cyclic response was simulated by employing a kinematic-hardening law that is related to the back stress^45^. Through the crystal-plasticity modeling, we know that the large lattice strain in the B2 phase is mainly ascribed to three factors: the B2’s small elastic moduli (Supplementary Table 3), the increasing stress in the B2 phase (Supplementary Fig. 9c), and more importantly, the martensitic transformation in the B2 phase. We can also conclude that this multicomponent B2 phase predominantly deforms by the {110}<001> cube slip mode typical of the NiAl-type B2 phase and the martensitic transformation.

### Microstructure evolution during cyclic deformation

**Fig. 4:**
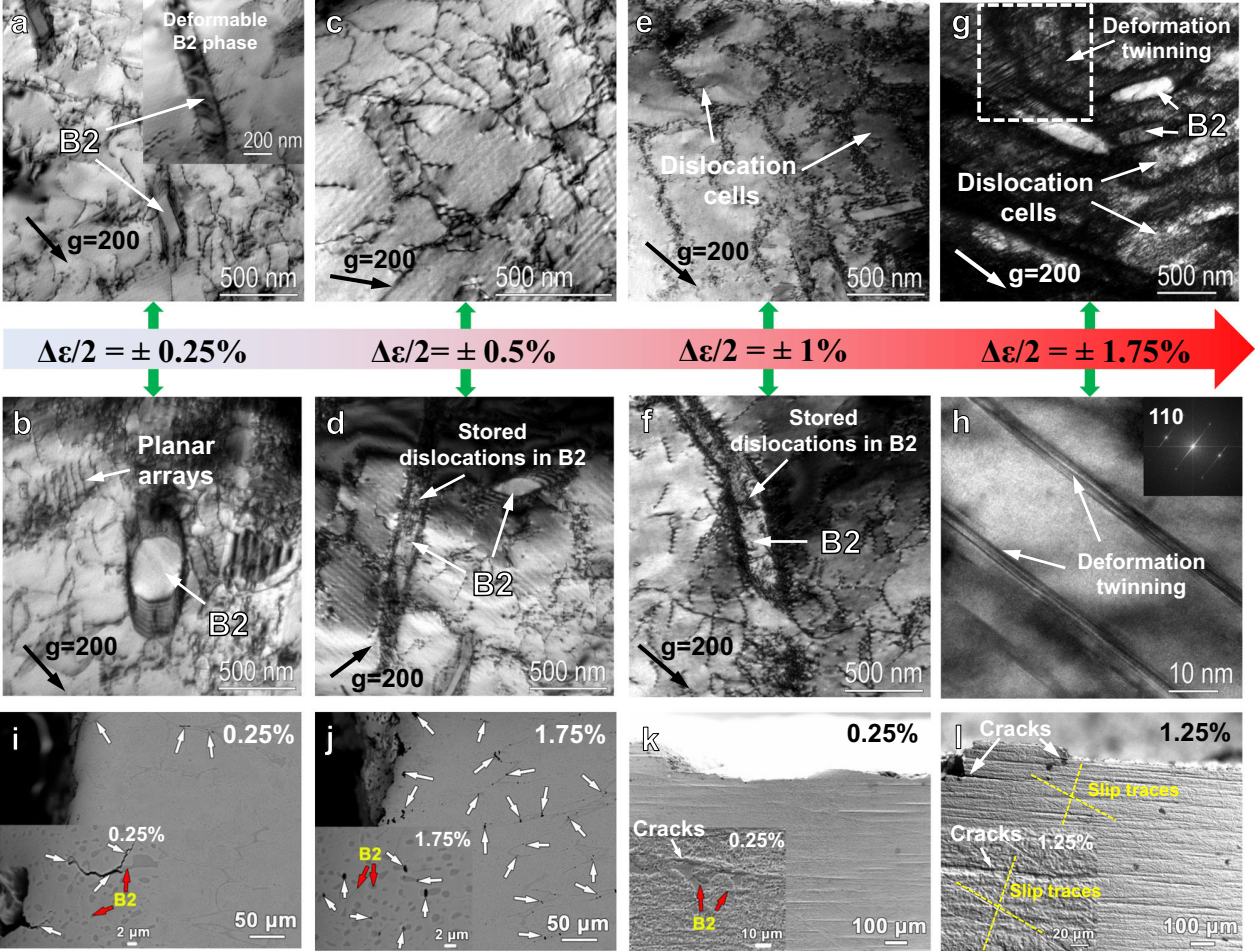
TEM and SEM characterized structural evolutions at different strain amplitudes. **a**, **b**; **c**, **d**; **e**, **f**; and **g**, **h** are the TEM bright-field (BF) images at the strain amplitudes of ±0.25%, ±0.5%, ±1%, and ±1.75%, respectively, showing the structural evolution of the cyclic response of deformation features (the inset figure in Fig. 5a) is the dark-field (DF) image, presenting the plastically-deformable B2 phase). **i**–**l** are the SEM images of the fractured samples at the strain amplitudes of ±0.25%, ±1.25%, and ±1.75%, exhibiting the crack-formation features at low and high strain amplitudes.

Figure 4a–h display the deformation features from the low strain amplitude of 0.25% to the highest strain amplitude of 1.75% after the LCF testing. At the low strain amplitudes (below 1%), the dislocation slips, including cross slips and planar arrays, prevailed (Fig. 4a–d). As the strain amplitude increased to 1%, dislocation cells were formed, resulting from the extensive cross slips of dislocations in the FCC matrix. No deformation twinning was observed at the strain amplitude of 1% after screening more than 20 grains, in line with the in situ neutron results (Supplementary Fig. 8). When the strain amplitudes further increased to 1.75%, elongated dislocation cells were found, separated by much denser and severely-tangled dislocations. Moreover, deformation nanotwins were found, as presented in the bright-field (BF) TEM image (Fig. 4g and Supplementary Fig. 10) and high-resolution TEM (HRTEM) (Fig. 4h). Note that the deformation twins formed in the severely-tangled dislocations region (highlighted by the dashed box in Fig. 4g). This is because the formation of deformation twinning requires a sufficient resolved shear stress to reach the critical stress of twinning^46^. The critical stress of twinning of the studied alloy is calculated as high as 1233 MPa at room temperature (RT) (Supplementary Note 3)^47^, leading to the difficulty in forming deformation twins during monotonic loading at RT (Supplementary Fig. 11). However, the deformation twinning formed during cyclic loading when the strain amplitude was larger than 1%, where the maximum stress was only about 733 MPa. It is because that the dislocations are more tangled and denser at high cyclic-strain amplitudes than those at low cyclic-strain amplitudes and during monotonic tension, leading to the local shear stress easily beyond the critical stress of twinning.

At all strain amplitudes, dense dislocations piled-up around the B2-phase boundaries, due to the strain incompatibility of the FCC and B2 phases. Upon the cyclic testing, the plastic deformation occurred firstly in the soft FCC matrix, leading to the great increase of the dislocation density by the multiplication and rearrangement of dislocation substructures in the FCC matrix. In contrast, the hard B2 precipitates acted as obstacles and resisted the plastic deformation, which hindered the further movements of dislocations, and the internal interphase stress developed, contributing to the cyclic strain hardening. As the cycle and cyclic strain increased, the plastic deformation propagated gradually into the hard B2 phase, as evidenced by the severely-stored dislocations in the B2 phase (Fig. 4 and Supplementary Fig. 10). Interestingly, the B2 phase also experienced eminent plastic deformation at low strain amplitudes (Fig. 4a, b and Supplementary Fig. 5a–c), implying that the martensitic transformation also occurs at the low strain amplitudes, as demonstrated by the ex situ HRXRD results on the strain amplitude of 0.25% (Supplementary Fig. 5b, c). The heterogeneous deformation between the hard B2 and soft FCC phases raised a localized stress concentration between their boundaries and caused microcracks. However, unlike the traditional binary NiAl B2 phase, the multicomponent NiAl-rich B2 phase can be subjected to more plastic deformation and martensitic transformation, which provided necessary accommodation between the FCC and B2 phases, resulting in the relaxation of the severe stress concentration and retarding the initiation of microcracks. As shown in Fig. 4i, very few microcracks were initiated at the low strain amplitude (0.25%), indicating the high fatigue-crack-initiation resistance, and thus, resulting in a longer fatigue life at low strain amplitudes of the studied HEA (Fig. 2e). Even though more cracks formed at high strain amplitudes, e.g., 1.75%, those microcracks mainly initiated and propagated near the larger band-like B2 precipitates with longer incoherent interfaces where the stress concentration cannot be effectively accommodated (Fig. 4j). However, near the fine B2 precipitates, the circle-like cracks were formed with blunted crack tips, suggesting that the fine B2 phase could act as efficient crack arresters to inhibit crack propagation and coalescence during cyclic deformation^48^. Thus, the fine ductile-transformable B2 phase enhanced the fatigue-crack-initiation resistance and improved the fatigue life.

### MC simulations

**Fig. 5:**
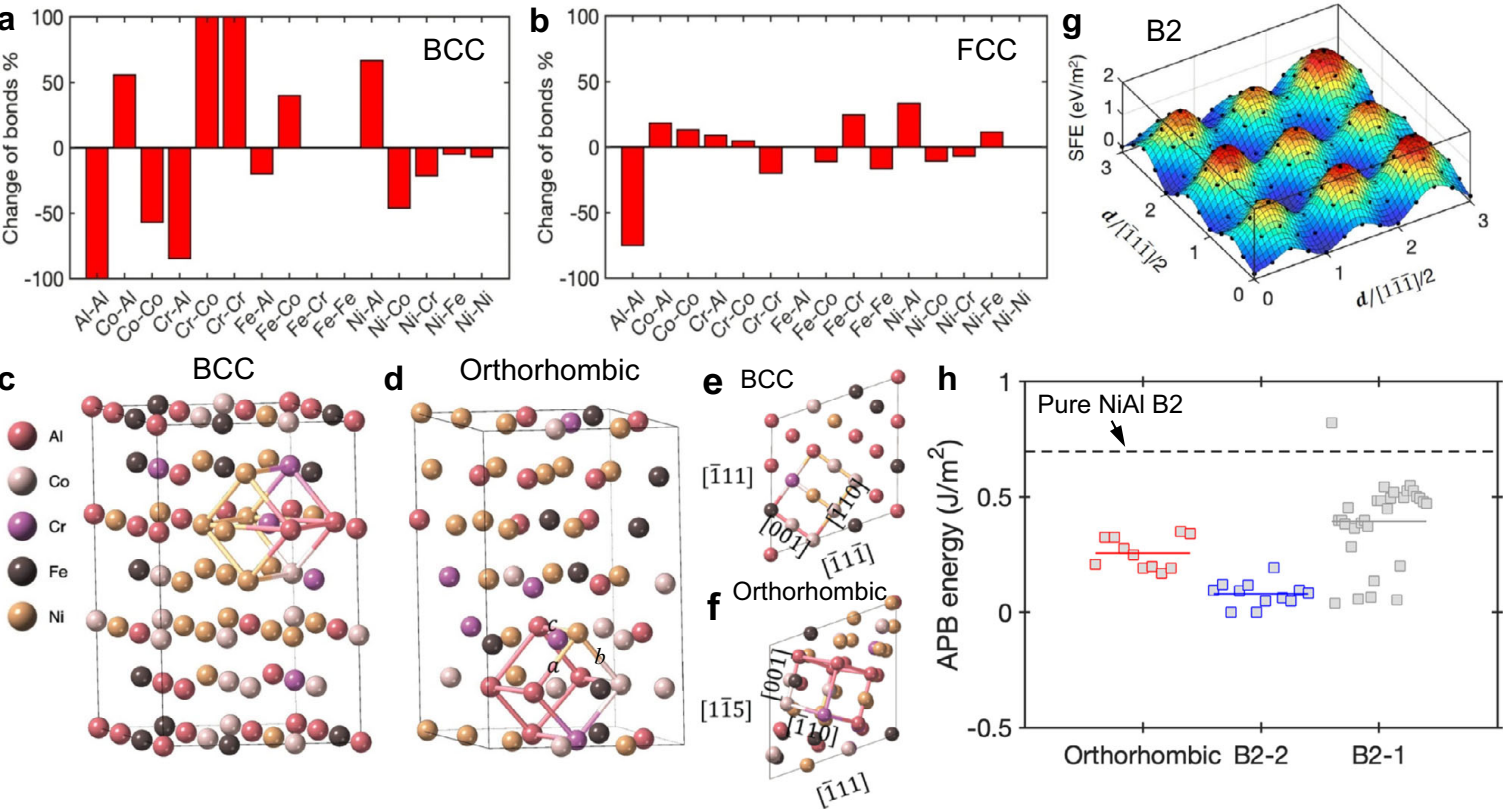
MC simulation results. **a**, **b** First nearest neighbor bonding environment change for BCC and FCC phases, respectively, after the MC simulation. The change is relative to the number of bonds in the initial configuration. **c**–**f** Structures of the precipitate phase before and after the MC simulation. **c** Initial BCC structure. **d** Final ordered orthorhombic structure (*a* = 3.014 Å, *b* = 2.902 Å, and *c* = 2.571 Å). **e** top view before MC. **f** top view after MC. **g** Generalized SFE distribution of the final B2 phase. **h** Comparison of APB energies of the B2 phase with different configurations. The dashed line indicates the stacking fault energy of a pure NiAl B2.

Further, first-principles calculations and MC simulations revealed the ductile-transformable character of the multicomponent B2 phase. The site occupancy of the B2 phase was determined by the changes in the first nearest neighbor bonding at the end of the MC simulation (Fig. 5a). The changes were relative and calculated, using Δ = (*n' – n*)/*n* × 100% where *n* is the number of the first nearest bonds of a given atom type, and *n'* is the corresponding number after the MC simulation. From Fig. 5b, we can see that a stronger site preference is in the BCC phase, while the FCC phase is more of a random solid solution, except that Al-Al bonds are strongly unfavorable. In the BCC phase, the largest decrease of bonds happens for Al–Al, Co–Co, Cr–Al, and Ni–Co bonds, indicating that Al and Co are confined to separate sublattices. Also, Cr and Al are on the same sublattice, and so are Ni and Co. Meanwhile, Al and Cr are on the same sublattice from the fact that Co–Al and Co–Cr bonds both increase. Also, Ni and Al are assumed to form the basis of the two sublattices. As for Fe, its occupancy can be deduced from the fact that the composition on either sublattice should add up to 50%. Therefore, for this material, the site occupancy can be determined as Al, Cr, and Fe on one sublattice and Co and Ni on the other.

During the MC simulation, the lattice vectors of the BCC phase were set to the experimental lattice parameters (Fig. 5c), but the atoms undergo obvious local distortions after relaxation (Fig. 5d). Due to the strong local distortions, a significant change in the lattice parameter happens. In the unit highlighted in Fig. 5e, f, we can measure that *a* = 3.014 Å, *b* = 2.902 Å, and *c* = 2.571 Å, an orthorhombic structure, indicating a possible martensitic transformation can occur in the B2 phase.

We also calculated the planar fault energies in this multicomponent B2 phase to reveal its strengthening effect and plastic deformability. Due to the K–S orientation relationship between FCC and B2 phases, we mainly focused on the stacking faults of the {110} planes in the B2 phase. In addition to the orthorhombic structure from the MC simulation, we considered two additional partially-ordered B2 structures for comparison. One has Ni and Co on one sublattice and the rest of the elements on the other sublattice (B2-1), as determined from the simulation. A second possible B2 phase (B2-2) for comparison has Ni and Fe on one sublattice and Al, Co, and Cr on the other, which is derived from the B2 compound, AlFe^49^. Unlike the FCC structure, the B2 structure does not have stable stacking faults where only full dislocations are allowed. Whereas for the ordered B2 structures, we have antiphase boundaries (APB) as an addition. Whichever case it is, only dislocations with Burgers vectors of ${b}_{1}=[1\bar{1}\bar{1}]/2$ or their equivalent are permissible for the slip in the B2 phase (Fig. 5g). Given the K–S relationship, we can easily see that the full dislocation in the FCC matrix is parallel to *b*_1_ or its equivalent, whereas Shockley partials of $\langle 112\rangle a/6$ kind are not. This feature means that the movement of these dissociated dislocations would be hindered by the B2 phase. The leading partial has to wait for the trailing partial to recombine before they can shear through the B2 phase, providing potential strengthening to the material. Fig. 5h presents the APB energies of the possible configurations of the multicomponent B2 and its transformed variant, an orthorhombic structure. The pure NiAl B2 has a high APB energy of 0.697 eV/m^2^, which is a commonly-accepted reason for the brittleness of traditional B2-strengthened materials. Obviously, all the possible configurations of the multicomponent B2 have lower APB energies than the pure NiAl B2 phase (Fig. 5h). The APB energy measures the level of difficulty of changing from one configuration to another. If a configuration has a very low or negative APB energy, it can easily transform to another stacking, indicative of less stability. On the contrary, it is harder to deform a configuration with higher APB energies, which in turn shows that the structure itself is more stable. Thus, we can conclude that the B2 phase with Ni and Co on one sublattice and other elements on the other is the most stable configuration due to its highest SFE. It is consistent with the configuration deduced from bond changes during MC simulations. Regardless of which configuration the B2 phase has, the addition of more elements lowers the APB energy, compared to that in the binary NiAl B2, which enables more plastic deformations.

## Discussion

**Fig. 6:**
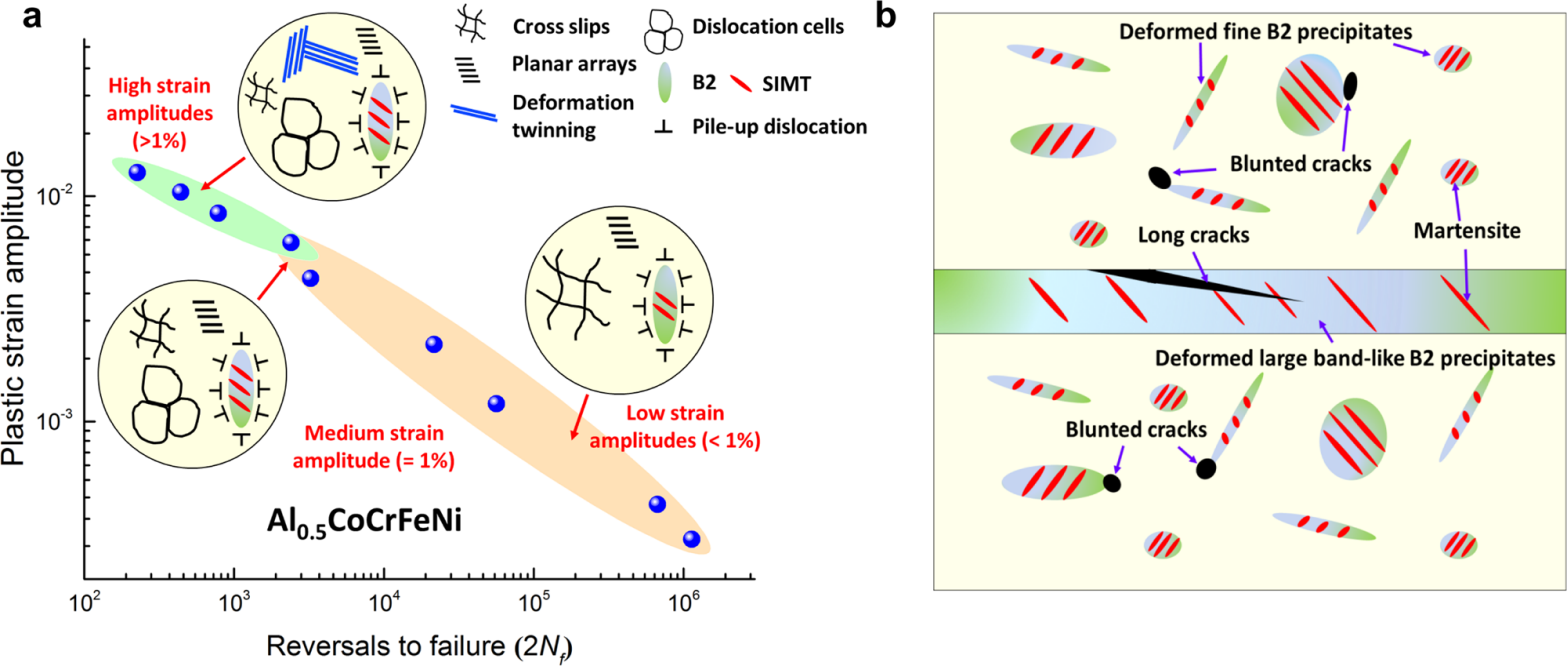
Schematics of the cyclic-deformation mechanisms and the microcrack-initiation behavior in the studied HEA. **a** Summary of dominated cyclic-deformation mechanisms of Al_0.5_CoCrFeNi at different strain amplitudes. **b** Schematic of the microcrack-formation mechanism during cyclic loading.

The ductile-transformable character of the multicomponent B2 phase plays a critical role in strengthening the fatigue-crack-initiation resistance, leading to the enhanced fatigue life at low strain amplitudes. Once the microcracks are initiated, the annihilation rate of dislocations is accelerated, resulting in cyclic softening and shortening the fatigue life. Another cyclic-softening source may be related to the SRO or the not high SFE (~49 mJ/m^2^)^50^ in this HEA, causing the slip planarity and leading to the strain localization^24^. In this alloy, Al–Ni and Cr–Fe are the possible SRO pairs in the FCC matrix, as suggested by the large increased Al–Ni and Cr–Fe bonds in the FCC phase (Fig. 5b). Once the SRO is broken down by the leading dislocation, the following dislocation faces a lower resistance to slip, contributing to the cyclic softening. Due to the presence of SRO, the planar slip tended to form, as manifested by the observed planar arrays (Fig. 4b). However, the slip planarity is not obvious at low strain amplitudes, compared to that at high strain amplitudes, by looking at the low-magnification surface feature (Fig. 4k, l). Moreover, the strain localization caused by the slip planarity did not result in obvious cracks. Instead, microcracks mainly initiated near the large band-like B2 phase. Both the formation of microcracks and the presence of SRO are responsible for the late-stage cyclic softening at low strain amplitudes (<1%). At high strain amplitudes (>1%), the deformation twinning occurred, which acted as barriers to hinder the further dislocation movement, and then notable cyclic hardening can be achieved to overcome the softening caused by the microcracks and SRO effect. Thus, the cyclic stability was observed after initial hardening at high strain amplitudes (≥1%) (Figs. 2b and 3e). The overall understanding of the evolution of cyclic-deformation mechanisms versus the strain amplitudes is summarized in Fig. 6a.

Our results demonstrate that fatigue life can be improved at low strain amplitudes by incorporating multicomponent ductile and transformable intermetallic precipitates, superior to other reported traditional intermetallic precipitates-strengthened alloys, and meanwhile, maintaining comparable fatigue life at high strain amplitudes. The fine ductile-transformable B2 phase effectively retards the initiation of microcracks due to the effects of stress relaxation and strain partitioning during plastic deformation and martensitic transformation (Fig. 6b), which are beneficial to relieve cyclic accumulation of damage caused by the deformation incompatibility between the B2 and FCC phases^51^. The ductile-transformable character of the multicomponent B2 phase results from its lower APB energy and strong local lattice distortions, making it easily transform to an orthorhombic structure. Such transformation can alleviate the need for five independent dislocation slip systems that are usually difficult to meet in traditional intermetallics by offering an alternative deformation pathway^52^. Thus, even though their size is large (up to ~5 μm), the B2 phase can still deform plastically, distinct from the traditional intermetallic precipitates that cannot bear too much plasticity in such large sizes. Certainly, once the B2’s size is too large, like the band-like B2 phase, it becomes easy to initiate microcracks due to longer incoherent interfaces (Fig. 6b and Supplementary Fig. 12). Nevertheless, the study suggests that the multicomponent B2 phase could not only strengthen the soft FCC matrix but also coordinate the overall plastic deformation during monotonic and cyclic loadings. Furthermore, the deformation twinning can also be induced during cyclic loading in a precipitation-strengthened HEA that is usually difficult to be seen during monotonic deformation at RT, providing additional strengthening for improving fatigue life. Our work provides a full picture to understand the cyclic-deformation mechanisms in the multicomponent B2 precipitates-strengthened HEA, and guides the design of fatigue-resistant alloys by introducing ductile-transformable multicomponent intermetallic precipitates that can be easily achieved by tuning HEAs’ compositions and thermomechanical processing.

## Methods

### Sample preparation

A large plate of Al_0.5_CoCrFeNi (~203 × 305 × 76 mm) has been fabricated, employing a vacuum-induction melting method. The large ingot was then hot rolled with a reduction of 60% at 1150 °C to reduce casting defects and refine grain sizes. After hot rolling, the samples were annealed at 1000 °C for 1 h to obtain a recrystallized microstructure and a certain amount of B2 precipitates.

### Mechanical tests

The specimens for the LCF tests with an 8-mm diameter and 16-mm gauge length were machined along the rolling direction, according to the American Society for Testing and Materials (ASTM) Standard, E606-04e^53^ with a dog-bone shape. After sample machining, the surfaces of the LCF samples were polished to 1200 grits. The monotonic tension and fully-reversed strain-controlled LCF tests were performed at RT, using an MTS Model 810 servohydraulic machine. The monotonic tension was performed at RT with a strain rate of 1 × 10^−3^ s^−1^. The LCF tests were conducted in a wide strain amplitude range from ±0.1% to ±1.75% with a triangular loading waveform under a continuous-loading condition. The LCF tests were performed at the strain rate of 1 × 10^−2^ s^−1^, and the initial loading is tension. All the strains were measured by an MTS extensometer.

### Microstructural characterizations

The studied alloys were characterized before and after LCF tests, using SEM, EBSD, EDS, and TEM techniques. The microstructure was characterized, by SEM, employing a Zeiss EVO scanning-electron microscope equipped with the backscattering electron (BSE), EDS, and EBSD detectors. The crystal structures of this multiphase alloy were identified by HEXRD with a wavelength of 0.117418 Å on the 11-ID-C beam line at the Advanced Photon Source (APS), Argonne National Laboratory. The specimens for SEM and EBSD were initially polished to mirror finish, using the 1200-grit SiC paper and, subsequently, vibratorily polished, employing the 0.05-μm SiC suspension for the final surface clarification. The TEM was performed on a ZEISS LIBRA 200 HT FE MC. Thin foils for the TEM observations were electrical-discharge machined (EDM) from the gauge section of ruptured samples. The thin foils converted into 3-mm-diameter disks for the TEM observations were prepared by twin-jet polishing, using an electrolyte consisting of 95% (volume percent) ethanol and 5% perchloric acid in a volume fraction at a temperature of −40 °C and an applied voltage of 30 V. The examination of many specimens, including foils from the undeformed section, exhibited that very few artifact dislocations were produced by this TEM sample-preparation technique.

### In situ neutron-diffraction measurements

In situ neutron-diffraction experiments subjected to tension and fully-reversed strain cycling were performed, using an MTS load-frame on the VULCAN Engineering Materials Diffractometer at the Spallation Neutron Source (SNS), Oak Ridge National Laboratory (ORNL)^54,55^. Low, intermediate, and high strain amplitudes of ±0.5%, ±1%, and ±1.75% were chosen for the in situ neutron-diffraction continuous measurements, respectively. The selected cycles for neutron diffraction were recorded with a strain rate of ~7.4 × 10^−6^ s^−1^, while other fatigue cycles were with a strain rate of 1 × 10^−2^ s^−1^. Moreover, the strain amplitude at ±1% was also measured, using the holding mode for the CMWP analysis. The collected data were reduced and analyzed, employing the VULCAN Data Reduction and Interactive Visualization softwarE (VDRIVE)^56^. The *hkl* plane-specific lattice strain of each respective phase is determined by ${\varepsilon }_{hkl}=({d}_{hkl}-{d}_{hkl}^{0})/{d}_{hkl}^{0}$, where ${d}_{hkl}^{0}$ and ${d}_{hkl}$ are the reference lattice *d*-spacing in the stress-free state and the lattice *d*-spacing during loading, respectively.

### Crystal-plasticity modeling

For completeness, a brief description was provided for the EVPSC model that incorporated martensitic transformation, whose detailed description can be found elsewhere^57, 58^. In the EVPSC model, the polycrystalline aggregate is treated as a homogeneous effective medium (HEM) and each grain as an ellipsoidal inclusion. Their interaction is obtained through the Eshelby’s solution. The model simultaneously solves series of nonlinear differential equations consisted of the elastic-viscoplastic single-crystal constitutive relations, the Eshelby’s solution for the interaction between each grain and the HEM, and the self-consistency criterion. The EVPSC model is well suited to interpret the measurements provided by neutron diffraction since the intrinsic characters of both techniques are consistent with each other.

The plastic deformation is accommodated by 12 $\{111\} < 1\bar{1}0 >$ slip systems in the FCC phase, 6 $\{111\} < 001 >$ slip systems, and 24 phase-transformation variants, $\{0.5\,0.5\,0.7071\} < 0.\bar{5}\,0.\bar{5}\,0.7071 >$ in the B2 phase. The threshold stress, ${\hat{g}}^{\alpha }$, of the deformation system, *α*, is given by

2 $${\hat{g}}^{\alpha }={g}_{0}^{\alpha }+({g}_{1}^{\alpha }+{\theta }_{1}^{\alpha }\varGamma )\left(1-\exp \left(-\frac{{\theta }_{0}^{\alpha }\varGamma }{{g}_{1}^{\alpha }}\right)\right)$$

where ${\theta }_{0}^{\alpha }$ and ${\theta }_{1}^{\alpha }$ are the initial and the asymptotic hardening rates, ${g}_{0}^{\alpha }$ and ${g}_{0}^{\alpha }+{g}_{1}^{\alpha }$ are the initial and the back-extrapolated critical resolved shear stresses (CRSS), respectively. $\varGamma$is the total accumulated shear strain over the shear strain, ${\gamma }^{\alpha }$, of all deformation systems (i.e., $\varGamma ={\sum }_{\alpha }\int |d{\gamma }^{\alpha }|$) in the grain. The evolution of the back stress, ${\dot{\tau }}_{b}^{\alpha }$, for the deformation system, *α*, is given by

3 $${\dot{\tau }}_{b}^{\alpha }={\xi }^{\alpha }{sgn}({\dot{\gamma }}^{\alpha })\mathop{\sum}\limits_{\alpha }{h}^{\alpha \beta }|{\dot{\gamma }}^{\beta }|-{\eta }^{\alpha }{\tau }_{b}^{a}\mathop{\sum}\limits_{\alpha }{h}^{\alpha \beta }|{\dot{\gamma }}^{\beta }|$$

where the first term is the linear kinematic hardening, and the second term is the dynamic recovery. ${\xi }^{\alpha }$ and ${\eta }^{\alpha }$ are the corresponding coefficients of the two terms. sgn is the sign function. ${\dot{\gamma }}^{\alpha }$ and ${\dot{\gamma }}^{\beta }$ are the shearing rates for slip systems of *α* and *β*, respectively. ${h}^{\alpha \beta }$ are the latent hardening coupling coefficients which empirically account for the obstacles on the  system, *α,* associated with the system, *β*, activity.

In the current model, the newly-formed martensite is treated as a hard inclusion, whose elastic constants and hardening parameters are set three times larger than those of the B2 phase. The elastic constants of the FCC and B2 phases that were determined, using the Kroner model^59^, are listed in Supplementary Table 3. Based on their lattice relation between the B2 and martensite phase, the volume change associated with the phase transformation is negligible, and hence, the primary strain is shear $({\gamma }^{PT}=4.3 \% )$.

The rate of the volume fraction, ${\dot{f}}^{\beta }$, of the phase transformation variant, *β*, is governed by a power-law relation

4 $${\dot{f}}^{\beta }={\dot{f}}_{0}{\left(\frac{\max ({\tau }^{\beta },0)}{{g}^{\beta }}\right)}^{\frac{1}{m}}$$

where ${\dot{f}}_{0}=0.001/s$ is a reference value. ${\tau }^{\beta }$ and ${g}^{\beta }$ are the resolved shear stress and threshold stress for the variant, *β*, respectively. *m* is the rate sensitivity.

Though the phase-transformation-induced strain is plastic, it simultaneously changes the lattice of the material. Therefore, in the calculation of the internal elastic strain, the contribution from the phase-transformation-induced strain is also considered.

### MC modeling

We use a MC method to determine the atomic arrangements in the B2 precipitates and the FCC matrix, respectively. We start with simulation cells representing systems of interest according to experimental results. We then find the most energetically-favorable atomic arrangement by applying a Metropolis MC^60^ algorithm, during which randomly-selected pairs of atoms are swapped. These swaps are accepted with the probability of $p=\,\min \,\{1,\exp (-\varDelta E/{k}_{{\mathrm{B}}}T)\}$, where Δ*E* is the change of the energy associated with the MC move, *k*_B_ is the Boltzmann constant, and *T* is the temperature. The energy and the configuration of this step are recorded, and the process will be repeated until convergence is achieved. The FCC cell consists of 6 {111} planes with a total of 54 atoms and a composition of 4Al–13Co–14Cr–13Fe–10Ni in terms of the number of atoms. The BCC cell also contains 54 atoms in the form of 6 {110} layers. The composition is 14Al–9Co–5Cr–7Fe–19Ni.

All energies are calculated, using the plane-wave-based density functional theory code, Vienna Ab initio Simulation Package (VASP)^61, 62^. The projector augmented wave method^63^ based pseudopotentials are used, and the exchange correlation is described by Perdew, Burke, and Ernzerhof^64^, with the valence electron configurations being s^2^p^1^ for Al, 3*d*^8^4*s*^1^ for Co, 3*d*^5^4*s*^1^ for Cr, 3*d*^7^4*s*^1^ for Fe, and 3*d*^8^4*s*^2^ for Ni. The Monkhorst-Pack *k*-point mesh^65^ for the Brillouin zone integration is 3 × 3 × 3. During relaxation, all degrees of freedom are allowed to relax, until forces on all atoms are smaller than 10^−2^ eV/Å. Due to the magnetic nature of these elements, spin polarization is enabled in our calculations.

## Supplementary information

Supplementary Information

## Data Availability

The data that support the findings of this study are available from the corresponding author upon reasonable request.
