# Supplementary material for: Enhancing fatigue life by ductile-transformable multicomponent B2 precipitates in a high-entropy alloy
Source: Nat Commun. 2021 Jun 11;12:3588. doi: 10.1038/s41467-021-23689-6 (PMC8196174; doi:10.1038/s41467-021-23689-6)
Supplement: Supplementary file 1 — Supplementary Information [file 41467_2021_23689_MOESM1_ESM.pdf]

Supplementary Information

Enhancing fatigue life by ductile-transformable multicomponent B2 precipitates in a high-entropy alloy

Rui Feng[1, 2], You Rao[3], Chuhao Liu[4], Xie Xie[1], Dunji Yu[2], Yan Chen[2], Maryam Ghazisaeidi[3], Tamas Ungar[5], Huamiao Wang[4], Ke An[2,*], and Peter. K. Liaw[1,*]

[1] Department of Materials Science and Engineering, The University of Tennessee, Knoxville, TN 37996, USA;

[2] Neutron Scattering Division, Oak Ridge National Laboratory, Oak Ridge, TN 37831, USA;

[3] Department of Materials Science and Engineering, The Ohio State University, Columbus, OH 43210, USA;

[4] State Key Laboratory of Mechanical System and Vibration, Shanghai Jiao Tong University, Shanghai, 200240, China;

[5] Department of Materials Physics, Eötvös University Budapest, PO Box 32, H-1518, Hungary;

* Corresponding authors: kean@ornl.gov and pliaw@utk.edu

Supplementary Figures

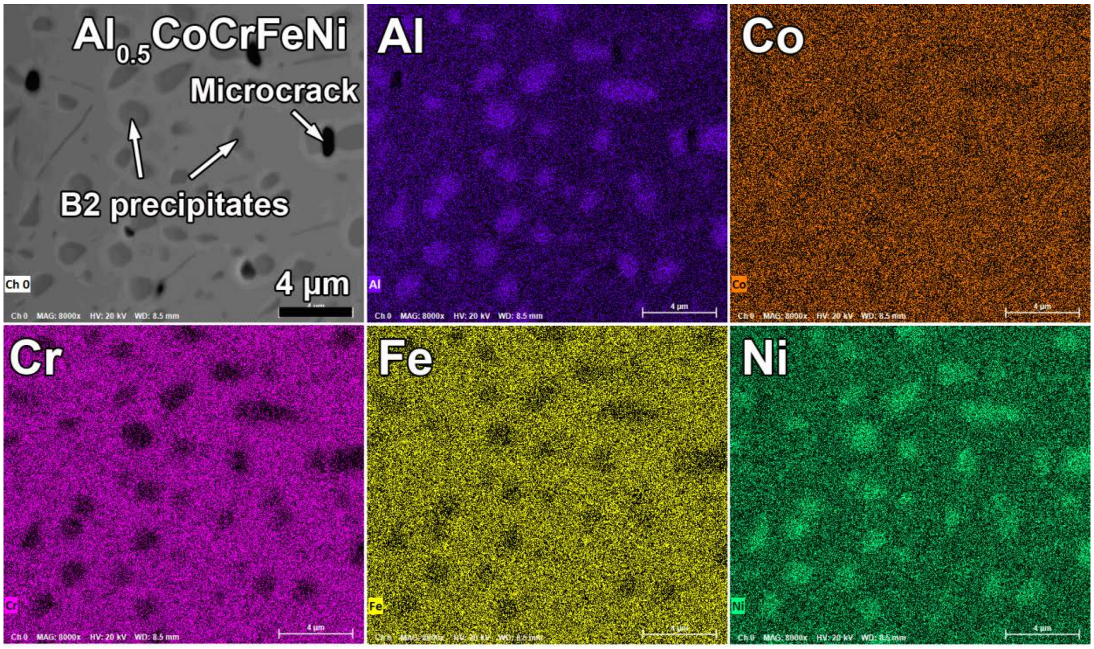

Supplementary Figure 1. Chemical composition distribution of the fatigue-fractured sample. EDS chemical compositional maps of the fatigue-fractured sample at the strain amplitude of ± 1.75%, exhibiting that the circle-like microcracks with blunted crack tips initiate at the fine NiAl-rich B2 precipitates.

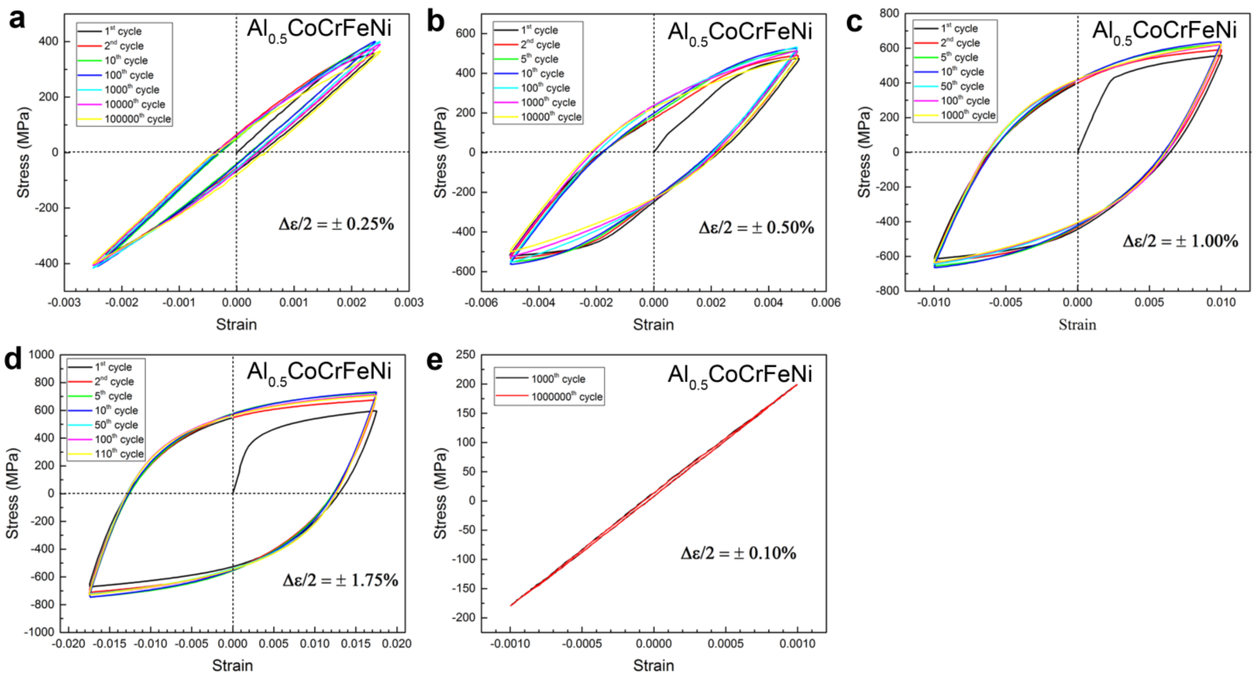

Supplementary Figure 2. Hysteresis loops of the $Al_{0.5}$CoCrFeNi HEA at selected typical numbers of cycles fatigued at different strain amplitudes. **a** Δε/2 = ± 0.25%. **b** Δε/2 = ± 0.5%. **c** Δε/2 = ± 1%. **d** Δε/2 = ± 1.75%. **e** Δε/2 = ± 0.1%. Note that the hysteresis loops at the strain amplitude of ± 0.1% after 1,000 and 1,000,000 cycles are still very small, suggesting a very small plastic deformation at the strain amplitude of ± 0.1%.

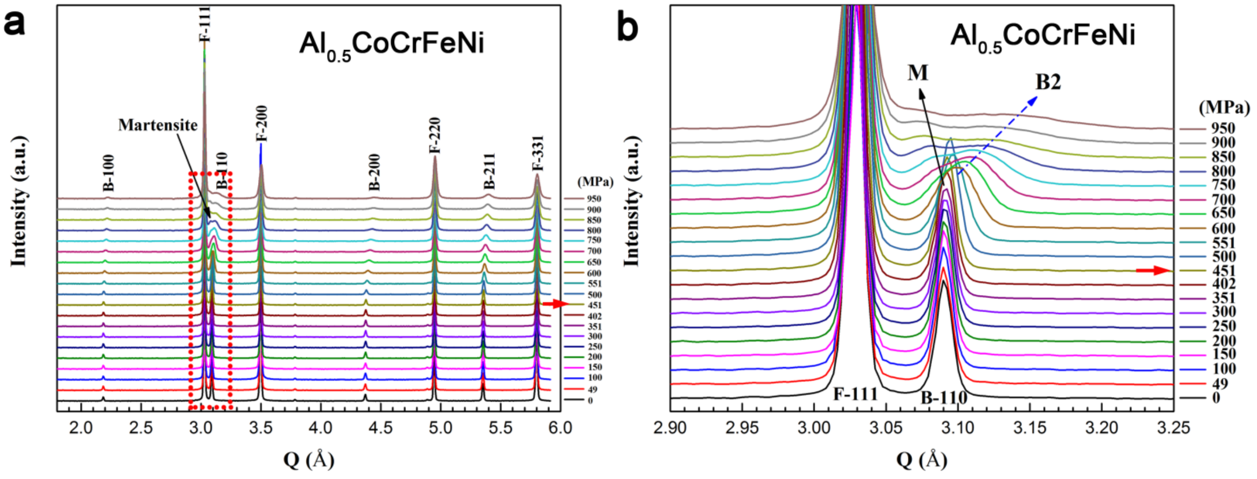

Supplementary Figure 3. Synchrotron XRD diffraction profiles as a function of the applied tensile stress. **a** Full patterns along the transverse direction (TD). **b** Enlarged B-[110] peaks along TD during the uniaxial tension (M denotes the martensite), showing the occurrence of the martensitic transformation upon the stress beyond 451 MPa (marked by the red arrow).

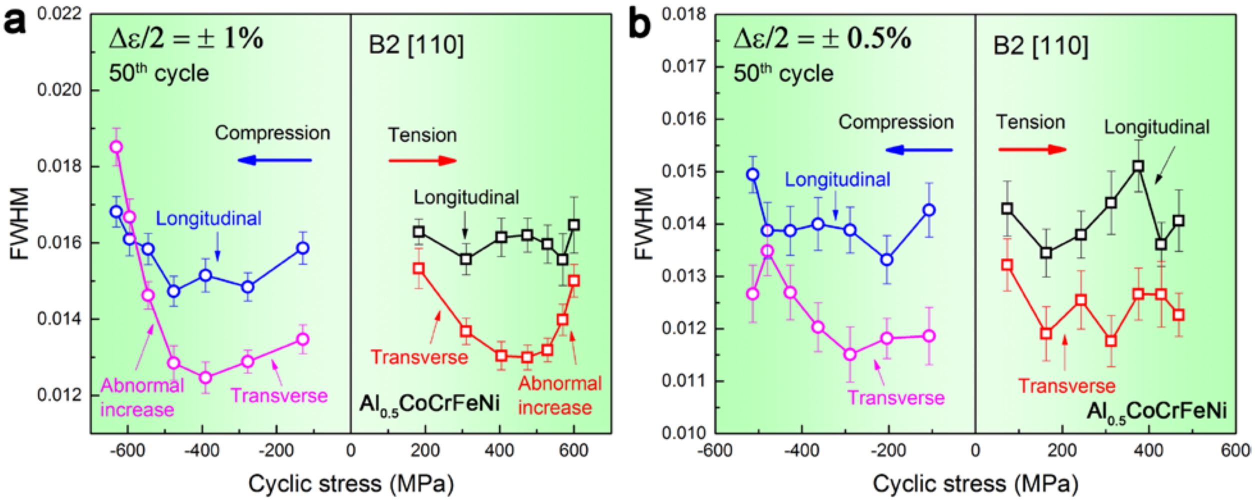

Supplementary Figure 4. Evolution of B2-[110] FWHM as a function of stress. The relationship of B2-[110] FWHM versus applied stress along the longitudinal and transverse directions at the 50th cycle under the strain amplitudes of ± 1% **a),** and ± 0.5% **b)**. The error bars are obtained from the uncertainties of the single-peak fitting on *hkl* diffraction peaks.

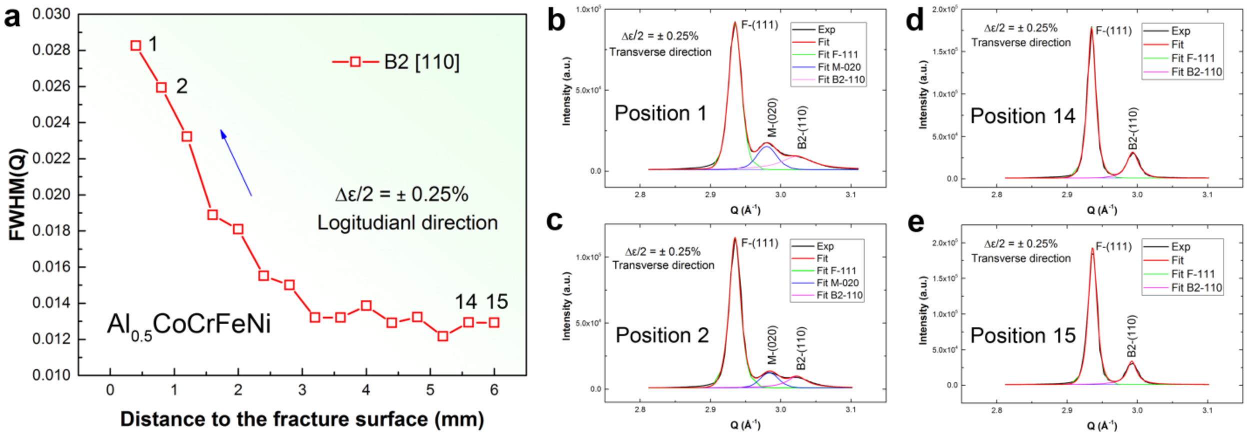

Supplementary Figure 5. *Ex-situ* HEXRD results on the LCF-fracture sample at the strain amplitude of ± 0.25%. a FWHM of B2-(110) along LD as a function of the distance to the fracture surface, indicating the dislocation density in the B2 phase is high during cyclic deformation. **b-e** Experimental and fitted HEXRD patterns of F-(111), B2-(110), and newly-formed martensite, M-(020) along TD. **b** and **c** show the formation of the orthorhombic martensitic structure at the positions of 1 and 2, respectively, in **a**, where is close to the fracture surface. **d** and **e** exhibit the absence of martensitic transformation at positions of 14 and 15, respectively, in Fig. **a**, where is far away from the fracture surface.

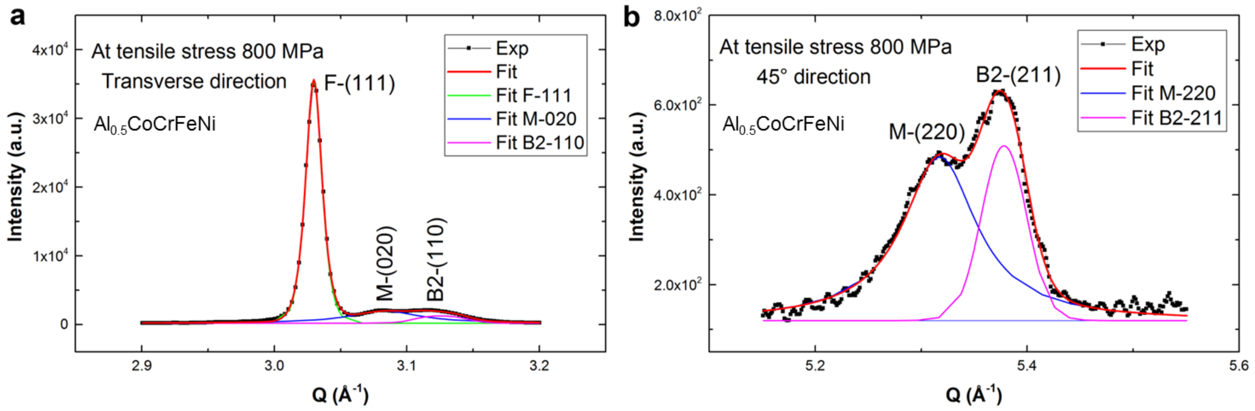

Supplementary Figure 6. HEXRD patterns at a uniaxial tensile stress of 800 MPa of the studied HEA for calculating the lattice parameters of the newly-formed martensite phase. a along the TD direction. **b** along the 45° direction.

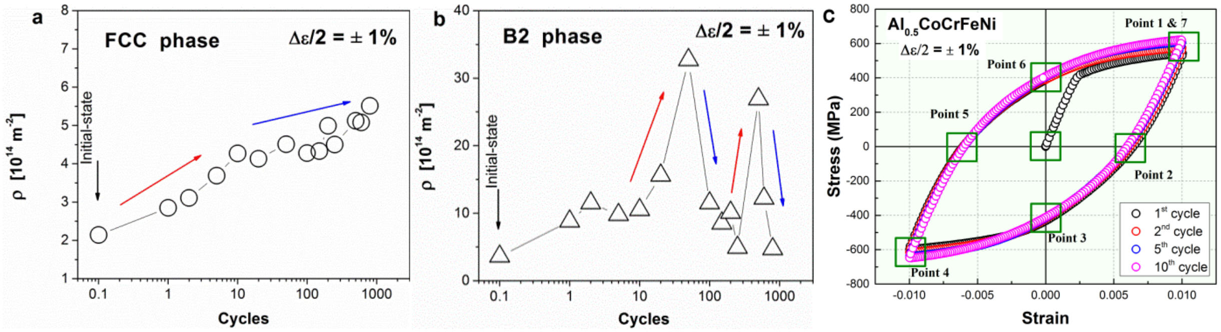

Supplementary Figure 7. The dislocation densities determined by the CMWP profile analysis. a The evolution of dislocation density in the FCC phase as a function of the number of cycles. **b** The evolution of dislocation density in the B2 phase as a function of the number of cycles. **c** Hysteresis loops of the $Al_{0.5}$CoCrFeNi HEA at the strain amplitude of ± 1%, where the marked points were selected for the 10-min. neutron-diffraction measurements used for the CMWP profile analysis in **a** and **b**.

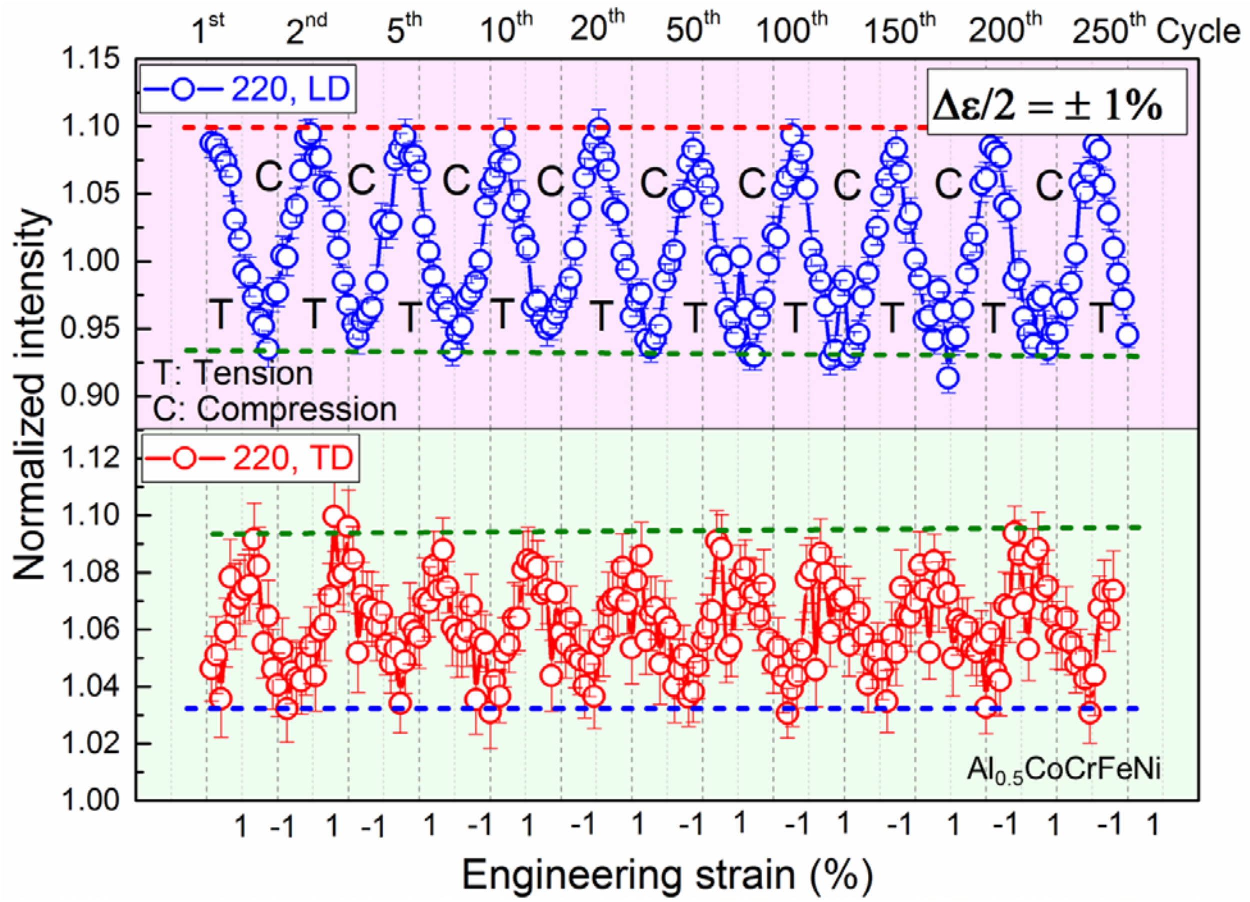

Supplementary Figure 8. *In-situ* neutron-diffraction measured intensity evolution as a function of applied strains at different fatigue cycles. The evolution of measured intensity as a function of applied strain at different fatigue cycles along longitudinal and transverse directions at the strain amplitude of ± 1%. The error bars are obtained from the uncertainties of the single-peak fitting on *hkl* diffraction peaks.

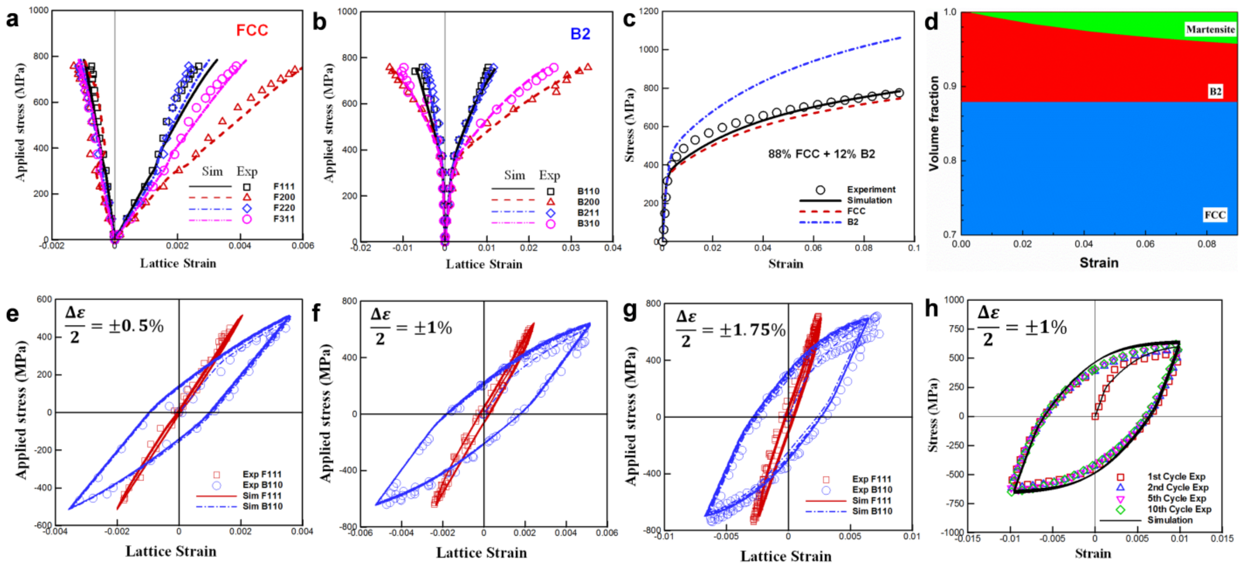

Supplementary Figure 9. EVPSC simulated results. a and **b** Comparison of experimental and simulated lattice strain versus applied stress of FCC and B2 phases under monotonic tension, respectively. **c** Experimental and simulated stress-strain curves of the studied HEA under tension. The phase-specific stresses obtained by the EVPSC model are also included, where the B2 phase is harder than the FCC phase and bears more stresses. **d** The respective volume fractions of the FCC, B2, and martensitic phases during tension. Phase transformation initiates at a strain around 1% (stress around 450 MPa). **e-g** Predicted and measured lattice strains of the HEA under cyclic loading with the strain amplitudes of e) ± 0.5%, f) ± 1%, and g) ± 1.75%. **h** Comparison of experimental and simulated macroscopic stress-strain curves during cycle-loading profiles of 1st, 2nd, 5th, and 10th cycles at the strain amplitude of ± 1%.

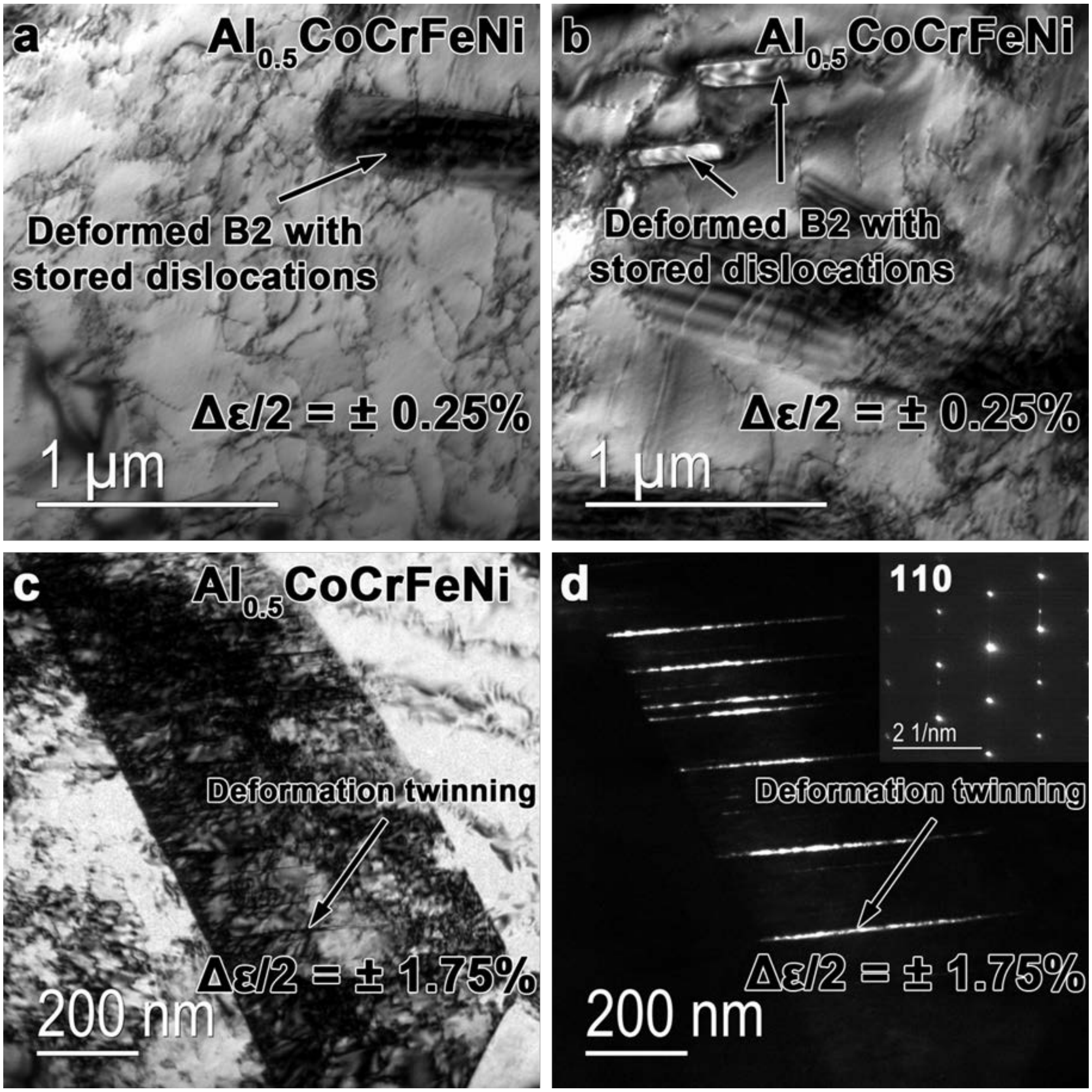

Supplementary Figure 10. TEM images of the typical microstructures of $Al_{0.5}CoCrFeNi$ after cyclic loading. a and **b** bright-field (BF) images at the strain amplitude of ± 0.25%, showing the deformed B2 precipitation with stored dislocations and the presence of dislocation slips. **c** and **d** BF and dark-field (DF) images at the strain amplitude of ± 1.75%, respectively, displaying the appearance of deformation twinning.

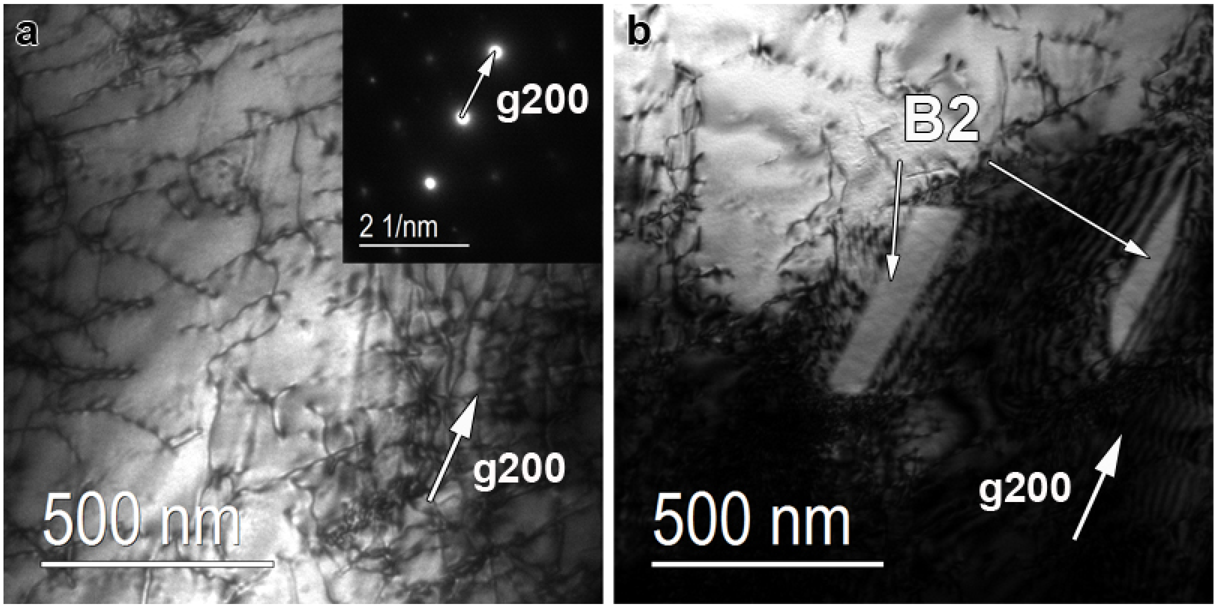

Supplementary Figure 11. TEM images of the uniaxial tension-tested $Al_{0.5}$CoCrFeNi HEA. **a** and **b** The dislocation features in the FCC phase regions where are far away from the B2 precipitates and near the B2 precipitates, respectively, after uniaxial tension of the $Al_{0.5}$CoCrFeNi HEA to 750 MPa, showing the absence of deformation twinning.

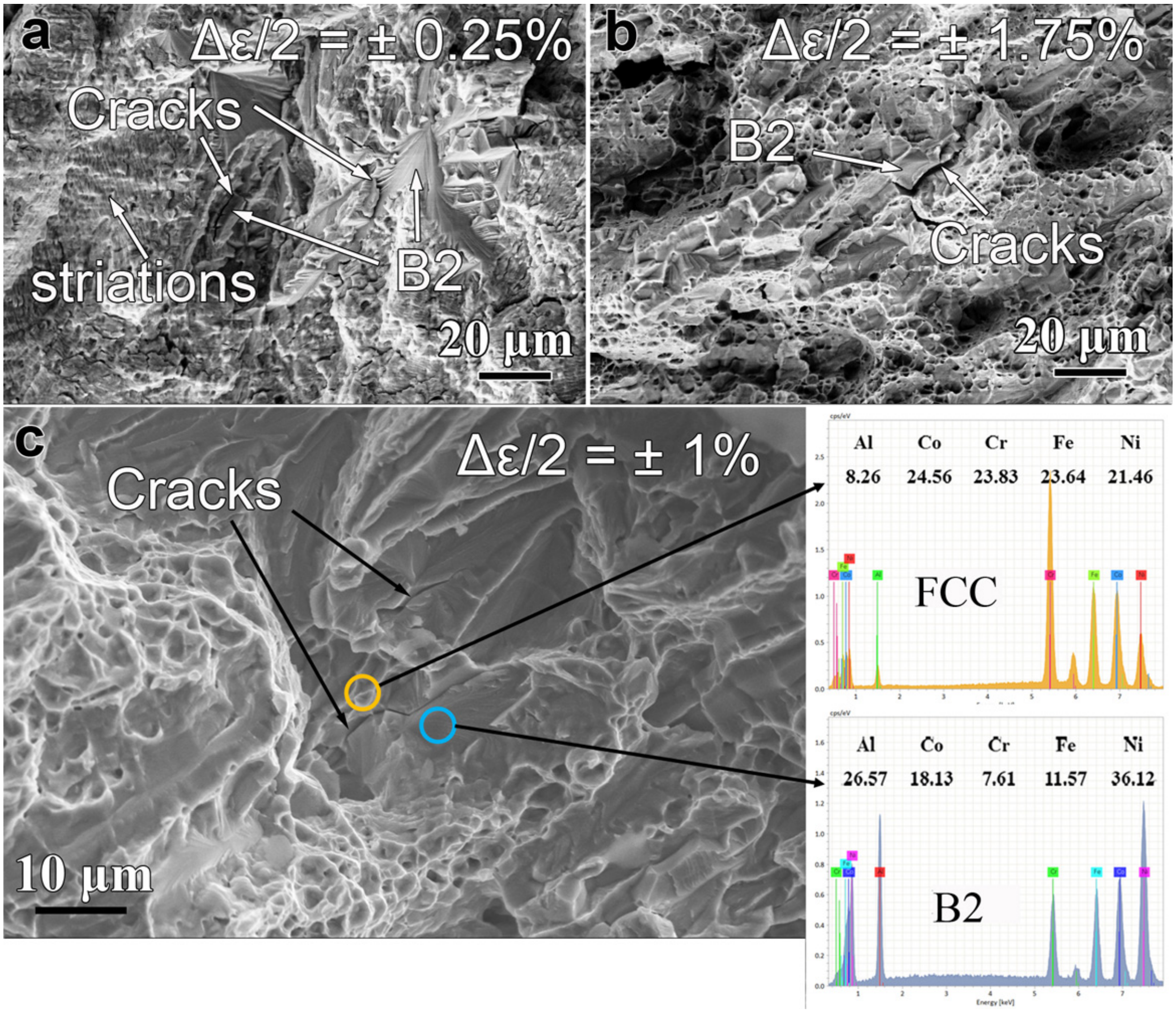

Supplementary Figure 12. The fractography of $Al_{0.5}CoCrFeNi$ after the low-cycle fatigue tests at different strain amplitudes. a at the strain amplitude of ± 0.25%. **b** at the strain amplitude of ± 1.75%. **c** at the strain amplitude of ± 1%, exhibiting that the cracks prefer to form near the large band-like B2 phase [the EDS results verify the FCC and coarse band-like B2 phases near the crack, the elemental concentrations in a unit of atomic percent (at%)].

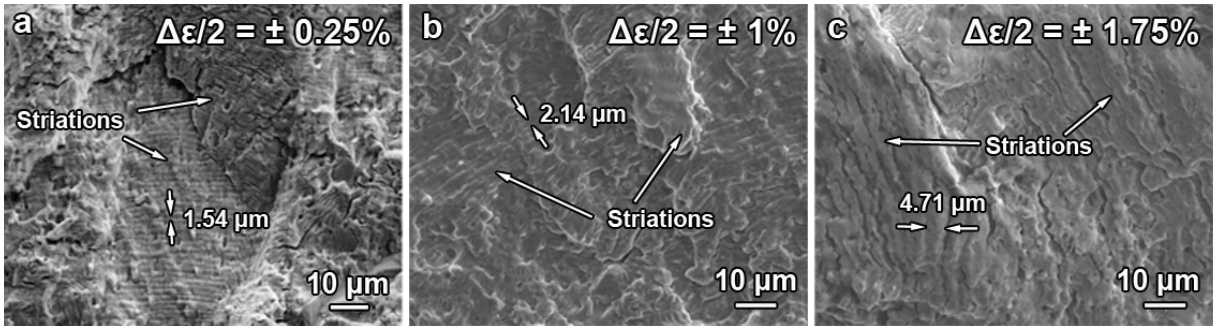

Supplementary Figure 13. Fractographies of the $Al_{0.5}$CoCrFeNi HEA near the edge of the fracture surface after the LCF testing at different strain amplitudes observed by SEM. a at $\Delta\varepsilon / 2 = \pm\ 0.25\%$. **b** at $\Delta\varepsilon / 2 = \pm\ 1\%$. **c** at $\Delta\varepsilon / 2 = \pm\ 1.75\%$, showing the increased crack-growth rate as the strain amplitude increases.

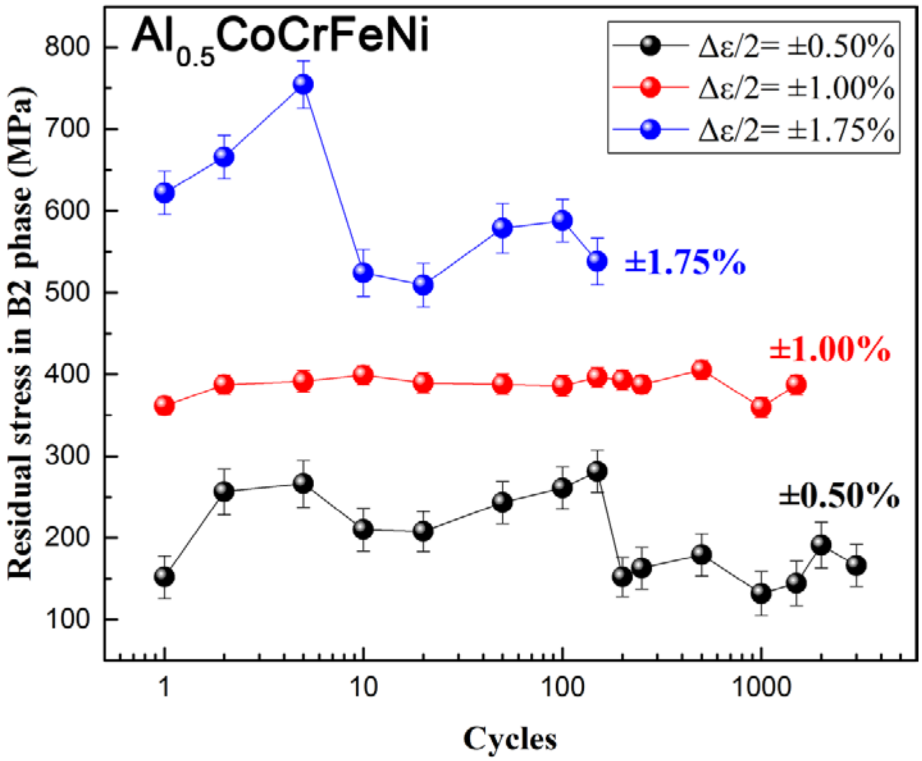

Supplementary Figure 14. Calculated residual stress in the B2 phase. The phase-specific residual stress in the B2 phase at the strain amplitudes of ± 0. 5%, ± 1%, and ± 1.75%. The error bars are obtained from the uncertainties of the single-peak fitting on *hkl* diffraction peaks.

Supplementary Tables

Supplementary Table 1. The fitting values of parameters shown in the strain-life equation

Parameters	$\sigma_f^{'}$ (MPa)	$\varepsilon_f^{'}$	b	c
Fitting values	1,315	0.10253	0.11414	0.36835

Supplementary Table 2. Predicted active slip modes and hardening parameters associated with the EVPSC-PT model, as in Eqs. (2) and (3) in the main text. Values for cyclic loadings are included in the brackets if different from those for monotonic tension.

Phase	Mode	g_0^α /MPa	g_1^α /MPa	θ_0^α /MPa	θ_1^α /MPa	$h^{\alpha\beta}$	ξ^α /MPa	η^α /MPa
FCC	$\{111\} < 1\bar{1}0 >$	110	75 (10)	1,500 (30)	350 (0)	1.4	$0(10^4)$	0(110)
B2	$\{110\} < 001 >$	500	250	7,050	0	1.4	0	0
	Transformation	250	1,550	15,050	0	1.4	NA	NA

Supplementary Table 3. Elastic constants of FCC and B2 phases in the $Al_{0.5}CoCrFeNi$ HEA, determined by the Kroner model[12, 13]

Elastic constant	C_{11}/GPa	C_{12}/GPa	C_{44}/GPa	Anisotropy
FCC	215.1	138.9	126.9	3.33
B2	200.3	121.0	91.8	2.3154

Supplementary Table 4. CMWP results of the $Al_{0.5}CoCrFeNi$-fatigue patterns at the strain amplitude of ± 1%

Cycles	0	1	2	5	10	20	50	100	150	200	250	500	600	800
ρ (FCC) [10^{14} m^{-2}]	2.14	2.85	3.11	3.69	4.27	4.13	4.51	4.28	4.33	4.99	4.5	5.12	5.08	5.55
ρ (B2) [10^{14} m^{-2}]	3.6	8.8	11.5	9.77	10.5	15.6	32.7	11.5	8.51	10.1	4.91	26.9	12.2	4.7

Supplementary Notes

Supplementary Note 1. Fatigue-life prediction

The fatigue life of materials is studied by the strain/stress-life equations, which is built, based on their stress or plastic strain. For the high-cycle fatigue (HCF) regime, the stress-based Basquin law (stress-life equation) is used to determine the fatigue life[1].

$$\Delta\sigma / 2 = \sigma_f^{'} (2N_f)^b \tag{1}$$

In the LCF regime, the plastic-strain-based Coffin-Manson law (strain-life equation) is widely used for the life prediction[2, 3].

$$\Delta\varepsilon_p / 2 = \varepsilon_f^{'} (2N_f)^c \tag{2}$$

Since the LCF tests contain both the elastic and plastic regimes, the Basquin and Coffin-Manson laws need to be combined for predicting the fatigue life. Therefore, the total fatigue-life prediction can be expressed as follows.

$$\Delta\varepsilon_t / 2 = \Delta\varepsilon_e / 2 + \Delta\varepsilon_p / 2 = \left(\frac{\sigma_f^{'}}{E}\right)(2N_f)^b + \varepsilon_f^{'} (2N_f)^c \tag{3}$$

where $\Delta\varepsilon_t / 2$ is the total strain amplitude; $\Delta\varepsilon_e / 2$ is the elastic-strain amplitude; $\Delta\varepsilon_p / 2$ is the plastic-strain amplitude; $\sigma_f^{'}$ is the fatigue-strength coefficient, MPa; *E* is the modulus of elasticity (174,150 MPa); N_f is the number of cycles to failure; b is the fatigue-strength exponent; $\varepsilon_f^{'}$ is the fatigue-strain coefficient; and c is the fatigue-ductility exponent. The fitted parameters for the total fatigue-life (Eq. 3) are given in Supplementary Table 1.

Supplementary Note 2. Convolutional-Multiple-Whole-Profile (CMWP) profile analysis

The CMWP evaluation of the $Al_{0.5}CoCrFeNi$-fatigue patterns at the strain amplitude of ± 1% was also performed[4-6]. In Supplementary Fig. 7 and Supplementary Table 4, it can be seen that the dislocation density is larger in the B2 phase than in the FCC phase, in line with the qualitative analysis in Fig. 3e. As the cycle increases, the dislocation density in the FCC phase increases greatly at the first 10 cycles. After the 10 cycles, the dislocations density in the FCC phase increases slowly and becomes saturated, similar to the results in Fig. 3e. Moreover, the dislocation density in the B2 phase shows two maxima, which is not clearly observed in Fig. 3e. The maxima might either be related to the reversible martensitic phase transformation or to changes in the texture.

Supplementary Note 3. Critical stress for deformation twinning

The critical stress of twinning with a specific grain size can be expressed as[7],

$$\sigma_T = m\frac{\gamma}{b_p} + \frac{k_T}{\sqrt{d}} \tag{4}$$

where m is the Taylor factor (m = 3.06), γ is the stacking-fault energy (SFE) (γ ≈ 49 mJ/m^2 [8]), b_p is the Burgers vector of a partial dislocation ($b_p = \frac{\sqrt{6}}{6}a = 1.467 \times 10^{-10}$ m), k_T is the Hall-Petch constant for twinning, and d is the grain size (~ 9 μm). For FCC-metallic materials, $k_T = 2 \times k_S$ is approximately estimated, based on its correlations with k_S, where k_S = 634 MPa μm$^{1/2}$ is the Hall-Petch constant for dislocation slips[9]. Therefore, the twinning stress is estimated as high as 1,233 MPa at RT.

Supplementary Note 4. Residual-stress calculation

The residual stress in a phase can be determined by the Hooke's law, based on the information concerning the lattice strains. Assuming the studied material to be isotropic, the three principal strain tensor components, ε_{11}, ε_{22}, and ε_{33}, are used to evaluate the stress-tensor components using the following equation[10]:

$$\sigma_{ii} = E_{hkl}[\varepsilon_{ii} + \nu_{hkl}\left(\varepsilon_{11} + \varepsilon_{22} + \varepsilon_{33}\right)/\left(1-2\nu_{hkl}\right)]/\left(1+\nu_{hkl}\right),\ i = 1,2,3 \quad (5)$$

where E_{hkl} and ν_{hkl} are the diffraction elastic constants and the Poission's ratio of the *hkl*-specific grains, respectively. It is usually believed that the stress can be accurately determined from the {211} lattice strains in a BCC structure and {311} or {111} lattice strains in an FCC structure, because these orientations are less sensitive to grain-orientation-dependent stresses[11]. Thus, the phase-specific residual stress in the B2 phase is calculated from the {211} lattice strains under the unloading condition at different cycles, as shown in Supplementary Fig. 14. The high residual stress in the B2 phase could explain why the reversible stress-induced martensite phase can be observed in the load-free state after cyclic loading.

Supplementary References

1. Basquin OH. The exponential law of endurance tests. *Proc. ASTM* 1910, **10:** 625-630.

2. Coffin Jr LF. A study of the effects of cyclic thermal stresses on a ductile metal. *Trans. ASME* 1954, **76:** 931-950.

3. Manson SS. *Behavior of materials under conditions of thermal stress*. National Advisory Committee for Aeronautics (1953).

4. Biermann H, Ungar T, Pfannenmüller T, Hoffmann G, Borbely A, Mughrabi H. Local variations of lattice parameter and long-range internal stresses during cyclic deformation of polycrystalline copper. *Acta Metall. Mater.* 1993, **41**(9)**:** 2743-2753.

5. Ungár T, Stoica AD, Tichy G, Wang X-L. Orientation-dependent evolution of the dislocation density in grain populations with different crystallographic orientations relative to the tensile axis in a polycrystalline aggregate of stainless steel. *Acta Mater.* 2014, **66:** 251-261.

6. Ungár T, Harjo S, Kawasaki T, Tomota Y, Ribárik G, Shi Z. Composite behavior of lath martensite steels induced by plastic strain, a new paradigm for the elastic-plastic response of martensitic steels. *Metall. Mater. Trans. A* 2017, **48**(1)**:** 159-167.

7. Sun SJ, Tian YZ, Lin HR, Yang HJ, Dong XG, Wang YH, *et al.* Transition of twinning behavior in CoCrFeMnNi high entropy alloy with grain refinement. *Mater. Sci. Eng. A* 2018, **712:** 603-607.

8. Li Q, Zhang TW, Qiao JW, Ma SG, Zhao D, Lu P, *et al.* Mechanical properties and deformation behavior of dual-phase $Al_{0.6}CoCrFeNi$ high-entropy alloys with heterogeneous structure at room and cryogenic temperatures. *J. Alloys Compd.* 2020, **816:** 152663.

9. Hou J, Shi X, Qiao J, Zhang Y, Liaw PK, Wu Y. Ultrafine-grained dual phase $Al_{0.45}CoCrFeNi$ high-entropy alloys. *Mater. Des.* 2019, **180:** 107910.

10. Guo P, Qian L, Meng J, Zhang F, Li L. Low-cycle fatigue behavior of a high manganese austenitic twin-induced plasticity steel. *Mater. Sci. Eng. A* 2013, **584:** 133-142.

11. Clausen B, Leffers T, Lorentzen T. On the proper selection of reflections for the measurement of bulk residual stresses by diffraction methods. *Acta Mater.* 2003, **51**(20)**:** 6181-6188.

12. Kröner E. Berechnung der elastischen Konstanten des Vielkristalls aus den Konstanten des Einkristalls. *Z. Phys.* 1958, **151:** 504-518.

13. Diao H, Ma D, Feng R, Liu T, Pu C, Zhang C, *et al*. Novel NiAl-strengthened high entropy alloys with balanced tensile strength and ductility. *Mater. Sci. Eng. A* 2019, **742:** 636-647.
